# The implication of autoantibodies in early diagnosis and monitoring of plasmonic photothermal therapy in the treatment of feline mammary carcinoma

**DOI:** 10.1038/s41598-021-89894-x

**Published:** 2021-05-17

**Authors:** Asmaa M. El-Rasikh, Haithem A. M. Farghali, Hisham A. Abdelrahman, Mostafa Elgaffary, Shaymaa Abdelmalek, Ibrahim A. Emam, Magdy A. Ghoneim, Salah A. Selim

**Affiliations:** 1 Department of Microbiology, Immunology, and Mycology, Faculty of Veterinary Medicine, Cairo University, Giza, 12211 Egypt; 2 Department of Surgery, Anesthesiology, and Radiology, Faculty of Veterinary Medicine, Cairo University, Giza, 12211 Egypt; 3 Department of Veterinary Hygiene and Management, Faculty of Veterinary Medicine, Cairo University, Giza, 12211 Egypt; 4 Department of Clinical Pathology, Faculty of Veterinary Medicine, Cairo University, Giza, 12211 Egypt; 5 Department of Biochemistry and Molecular Biology, Faculty of Veterinary Medicine, Cairo University, Giza, 12211 Egypt

**Keywords:** Biochemistry, Biological techniques, Cancer, Chemical biology, Immunology, Molecular biology, Biomarkers, Medical research, Oncology, Nanoscience and technology

## Abstract

Feline mammary carcinoma (FMC) shows great similarities to human breast cancer in the cellular and molecular levels. So, in cats as in humans, the role of immune responses is indicated to detect and follow up the development of tumors. As a new breast cancer therapeutic approach, Plasmonic Photothermal Therapy (PPTT) is an effective localized treatment for canine and feline mammary-carcinoma. Its systemic effect has not been inquired yet and needs many studies to hypothesis how the PPTT eradicates tumor cells. In this study, it is the first time to detect (P53, PCNA, MUC-1, and C-MYC) feline autoantibodies (AAbs), study the relationship between PCNA AAbs and mammary-tumors, and investigate the effect of PPTT on the humoral immune response of cats with mammary-carcinoma through detection of AAbs level before, during, and after the treatment. The four-AAbs panel was evaluated in serum of normal and clinically diagnosed cats with mammary tumors using Enzyme-Linked Immunosorbent Assay. The panel showed 100% specificity and 93.7% sensitivity to mammary tumors. The panel was evaluated in PPTT monotherapy, mastectomy monotherapy, and combination therapy. PPTT monotherapy decreased AAbs level significantly while mastectomy monotherapy and combination therapy had a nonsignificant effect on AAbs level.

## Introduction

Cats, as companion animals, share similar environmental risk factors as humans. The feline mammary tumor is the counterpart of human breast cancer and this is due to the great resemblance in the late age of onset, incidence, histopathologic features, biological behavior, and pattern of metastasis. This provides an essential conceptual tool for basic and clinical research, grantees a better understanding of breast cancer biology, and consequently establishing an animal patient model for studying breast cancer microenvironments, systemic reflections, and targeted therapies^1^.

The interaction between the immune system and cancer cells proceeds in three phases: elimination; equilibrium; and escape, which are referred to as the “three Es” of cancer immunoediting. During the ‘elimination’ phase, the immune system may succeed in destroying all tumor cells. If that does not happen, it may still be able to control tumor growth but not eradicating it. This phase is referred to as the ‘equilibrium’ phase. Finally, due to selection pressure from the immune system, some cancer cells develop enough resistance that they can escape the immune system, leading to a failure of immune-mediated cancer control which is called the ‘escape’ phase^2^. The release of proteins from tumors triggers an immune response in cancer patients. These tumor antigens or also called tumor-associated antigens (TAAs) arise from several mechanisms including tumor-specific alterations in protein expression, mutation, folding, degradation, or intracellular localization. Responses to most tumor antigens are rarely observed in healthy individuals, making the response itself a biomarker that betrays the presence of underlying cancer. It is not known if the antibodies to tumor-derived proteins reflect underlying immunosurveillance of cancer or have an impact on the clinical outcome of the disease^3^. The real usefulness of the TAAs as circulating tumor markers in the management of breast cancer has been questioned because of the low diagnostic sensitivity for the early disease before the appearance of clinical manifestation and prior recurrence or even metastasis. Moreover, their role in monitoring treatment responses through different stages of tumorigeneses, particularly after completion of therapy, remained unavailable^4^.

Circulating antitumor markers (AAbs) exhibit increased levels in very early cancer stages in sera prior TAAs can be detected as their production precede clinical confirmation of a tumor by several months or years^5^. AAbs that arise against the TAAs are present in the circulation of people with various forms of a solid tumor before TAAs can be detected, and these molecules can be measured up to 5 years before symptomatic disease^6^. Among 196 specific tumor-associated AAbs, the most frequently studied with a diagnostic value was the P53 antibody followed by autoantibodies against MUC-1, HER2, cyclin B1, NY-ESO-1, HSP60, and C-MYC. Combinations of tumor-associated AAbs showed higher diagnostic sensitivity than individual autoantibodies but single autoantibodies usually showed higher specificity^7^. Until now, a wide range of AAbs has been identified. Although several studies present hopeful preliminary results, there is a need to validate AAbs' signature for biomarker researches in the clinic^8^.

The p53 protein is involved in several critical pathways including cell cycle arrest, apoptosis, DNA repair, and cellular senescence, which are essential for normal cellular homeostasis and genome integrity maintenance. Alteration of the TP53 gene or posttranslational modification in p53 protein can alter its response to cellular stress. The molecular archaeology of the TP53 mutation spectrum generates hypotheses concerning the etiology and molecular pathogenesis of human cancer^9^. Mucin 1, cell surface-associated (MUC-1) also called polymorphic epithelial mucin (PEM) and epithelial membrane antigen (EMA) is a member of the mucin family which is a large, heavily transmembrane glycoprotein. The MUC-1 is generally expressed at low levels by normal simple secretory epithelial tissues, its overexpression is often associated with most carcinomas and in particular by breast cancers, and correlates with high metastatic and poor survival^10^. The MYC is a family (C-MYC, L-MYC, and N-MYC) of regulator genes and proto-oncogenes that code for transcription factors as a nuclear phosphoprotein that plays a role in cell cycle progression, apoptosis, and cellular transformation which regulate up to 15% of all human genes. Amplification of these genes is frequently observed in numerous human and animal cancers^11^. The C-MYC function is associated with specific molecular subtypes of breast cancer, its overexpression confers resistance to therapy and its activation has been widely reported in breast cancer progression^12^. Proliferating cell nuclear antigen (PCNA) is a non-histone nuclear protein associated with mitotic activity and tumor grade^13^.

Plasmonic Photothermal Therapy (PPTT) is a cancer therapy in which gold nanorods are locally injected into the tumor before exposure to near-infrared light causing localized cell death (apoptosis) which applied in dogs and cats suffering from naturally occurring mammary gland carcinoma by professor Mostafa A El-Sayed group and the treated cases showed no recurrence or metastasis for one year after treatment^14,15^. However, the mechanism by which PPTT induces complete ablation of small tumors and immune mechanisms underlying the prevention of in situ recurrence and distant metastasis induced by local PPTT therapy are unknown also the complex interaction between PPTT, breast cancer, and the immune system. Usually innate and adaptive immunity play a key role in the elimination of breast cancer. Adaptive immunity exerts its anticancer activity through the production of AAbs against the TAAs and cytotoxic effector CD8 + lymphocyte triggered by cell-mediated immune response^2^. However the role of AAbs in protecting against breast cancer remains elusive, but their validity in diagnosis, early diagnosis, prognosis, predictions, and monitoring of treatment response acquire raising interest and numerous publications reported the validity of AAbs assay in early diagnosis of breast cancer^16^.

The main target of the present investigation was to investigate the validity of AAbs assay in early diagnosis, prognosis, prediction, and monitoring of different treatments (PPTT monotherapy, surgical intervention only (Radical mastectomy), and combination treatment of surgery and PPTT.

## Methods

### Animals

The animals were admitted to the clinic of the Department of Surgery, Anesthesiology, and Radiology, Faculty of Veterinary Medicine, Cairo University, Egypt from August 2018 to February 2020. All cats did not receive any treatment for mammary tumors before. Mammary tumors were diagnosed through physical examination of cats. The initial tumor dimensions were measured in all animal groups using calipers. Additional measurements of tumor dimensions were made with each session of PPTT treatment. Cats were also examined by X-ray (Fisher, Berlin, Germany). The radiographic setting factors were 58–70 kVp, 10 mAs, and a 90 cm focal spot film distance. The radiographic exposures were conducted dorsoventrally and right laterally. The cats were divided into 5 groups which included group 1 (H: clinically healthy cats), group 2 (TN: tumor without treatment), group 3 (TP: tumor treated with PPTT monotherapy), group 4 (TS: tumor treated with mastectomy only) and group 5 (TSP: tumor treated with a combination of surgery and PPTT.) (Table 1).

**Table 1:** Animal groups.

Group no	Group name	Animal groups	Cases no	Lung metastasis
1	H	Apparently healthy cats	11	0
2	TN	cats with variable sizes of the mammary tumor and did not receive any treatment for the tumor	15	5
3	TP	cats bearing mammary tumors smaller than 10 cm^2^ treated with PPTT monotherapy	10	2
4	TS	cats bearing mammary tumors larger than 10 cm^2^ treated with surgery only	3	1
5	TSP	cats bearing mammary tumors larger than 10 cm^2^ treated with the combination of surgery and PPTT	6	2
		Total animal population	45 cat	10 cats

(TN) tumor-no treatment, (TP) tumor-PPTT alone, (TS) tumor-surgery alone, (TSP) tumor-surgery, and PPTT.

### Treatments

In the TP group, the number of PPTT cycles was correlated to the size of the mammary tumor with two weeks interval between each session^14^. Gold nanorods (AuNRs) with an average size of 27 (± 5) × 6 (± 1) nm (length × width) coated with Methoxy polyethylene glycol thiol (mPEG-SH) and Arg − Gly − Asp (RGD) peptides and 808 nm diode laser with a power of 0.5 W/cm^2^ and a spot size of around 5.6 mm^2^ were kindly gifted by prof. Mostafa A. El-Sayed; Laser Dynamics Laboratory, School of Chemistry and Biochemistry, Georgia Institute of Technology, USA. In the TS group, the mastectomy operations were performed as previously described^17^. Mastectomy operations were conducted by members of the surgery, anesthesiology, and radiology department of the Faculty of Veterinary Medicine, Cairo University. Under general injectable anesthesia, each animal was pre-medicated with atropine sulfate (1%, 0.05–0.1 mg/kg b. wt.; ADWIA Co. S.A.E., Cairo, Egypt) and xylazine HCl (1 mg/kg b. wt.; Xyla-Ject 2%, ADWIA Co. S.A.E.), and then anesthesia was induced using ketamine HCl (10–15 mg/kg b. wt.; Ketalar, Sigma-Aldrich Co.) and maintained by ketamine HCl^18^. In the TSP group, the treatment was performed according to previously described^15^.

### Samples

Serum samples were collected from cats treated with PPTT monotherapy (TP) before, during, and after the course of treatment with two weeks intervals between each sample. While the serum samples from cats treated with mastectomy only (TS) or combination therapy (TSP) were collected before surgery and within one month and a half after the surgery. All collected serum samples were immediately stored at − 40 °C until use.

### Checkerboard titration method

The first step was the standardization of indirect Enzyme-Linked Immunosorbent Assay (ELISA) by checkerboard titration as described in previous studies^19–22^ using the following recombinant proteins as coating antigens: TP53 (ABIN1046804), MUC1 (ABIN1877158), MYC (ABIN2130698), and PCNA (ABIN622005). All the antigens were purchased from Antibodies Online (https://www.antibodies-online.com/) (Germany) and they are of human origin. Antigens were subjugated to Lasergene software version 3. 18 (DNAStar, Madison, WI) analysis to evaluate their identity % with feline antigens (UniProtKB/ Swiss- Prot: P41685.1**,** NCBI Reference Sequence: XP_023103255.1, UniProtKB/ Swiss- Prot: P68271.1, and NCBI Reference Sequence: XP_003983789.1. The checkerboard titration was conducted to optimize antigen concentration and serum dilution. Antigen concentrations were 50, 25, 12.5, 6.25, 3.125, 1.5625, 0.78125 ng/well and serum dilutions were 1/40, 1/80, 1/160, 1/320, 1/640, 1/1280, 1/2560, 1/5120, 1/10,240, 1/20,480.

### ELISA

The optimal antigen concentration and serum dilution were then used for all subsequent ELISA tests and applied according to previously described in^23–32^. Coating of 96 well Immulon 4HBX Microtiter plates (cat. no. 3855; Thermo Fisher Scientific, USA) with antigen diluted in carbonate bicarbonate (SERVA Electrophoresis GmbH, Germany) buffer for 2 h at 37 °C then overnight at 4 °C. Washing four times with Phosphate Buffered Saline with 0.05% Tween 20, pH 7.4 (PBST) (SERVA Electrophoresis GmbH, Germany). Blocking the wells with 100 µl/well by 5% skimmed milk for 2 h at 37 °C. Washing then adding 100 µl of diluted serum samples in a duplicate manner and incubated for 90 min at 37 °C with shaking. Washing and adding of 100 µl per well of diluted Goat anti-feline IgG (H + L) secondary antibody horseradish peroxidase (HRP) conjugated (cat. no. PA1-84,673; Thermo Fisher Scientific, USA) according to manufacture instruction (1/10,000) incubated for 90 min at room temperature with shaking. Washing seven times with PBST and adding 50 µl/well of UltraTMB (3, 3’, 5, 5’-tetramethylbenzidine) coloring reagent (cat. no.34028; Thermo Fisher Scientific, USA) according to manufacture instruction. Stopping the reaction after 15 min with 50 µl/well of 2 M Sulfuric acid (ADWIC, Egypt) and the absorbance was read immediately at 450 nm BioTek ELX808IU ™ microtiter ELISA reader (BioTek, Winooski, VT, USA).

### Statistical analyses

The receiver operating characteristic (ROC) analysis was performed in SigmaPlot v14.0 (Systat Software, San Jose, CA, USA) using mammary tumor status (with tumor vs not tumor) as the binary state classification variable and AAbs values on a continuous scale as the test variable to determine the area under the curve (AUC) which is an important measure of the accuracy of the diagnostic marker. Pairwise comparisons of AUC among the four studied markers were performed using Chi-square (*χ*^2^) tests. On ROC curves, sensitivity, specificity, positive likelihood ratio (LR +), negative likelihood ratios (LR −), and Youden’s index (YI) were used to evaluate the diagnostic performance of all AAbs, and these parameters were based on the methodology provided in the Epidemiology textbook^33^. The optimal cut-off values on the ROC curves were determined from YI.

Kaplan–Meier survival analyses were performed for estimation of median survival times and survival probabilities of cats in each treatment group and cats with or without metastasis. Log-rank tests were used to compare the survival probability among treatments and to evaluate the effect of metastasis on survival probability.

For each marker, a two-way repeated-measures analysis of variance (ANOVA) test was used to compare between first and last marker values within each treatment group (TS, TP, and TSP). At any sampling event, the total tumor area (mm^2^) per cat was calculated. A one-way repeated-measures analysis of covariance (ANCOVA) test was used to compare all measured values for each marker among treatment groups while accounting for the effect of total tumor area as a covariate.

To evaluate the efficiency of studied markers in predicting the occurrence of tumor progressive events, a two-way repeated-measures ANOVA test was used for each marker to compare the predictive and pre-predictive values among treatment groups. The evaluated tumor progressive events were secondary, recurrent tumor, and metastasis. The term “predictive” value refers to the last marker value before the appearance of the tested event while the “pre-predictive” refers to the last marker value before the predictive value.

For the repeated measures tests, data were blocked by cat ID. The Shapiro–Wilk test was utilized for normality analysis of the variables. The Tukey's Studentized Range (HSD) test was used for posthoc analysis. All *p* values less than 0.05 were considered statistically significant. All data were presented as the mean ± standard error of the mean (*SE*). Analyses were performed with SAS version 9.4^34^.

### Ethical approval

The study was carried out in compliance with the ARRIVE guidelines^35,36^. All animals were handled following the Association for Assessment and Accreditation of Laboratory Animal Care and Office of Laboratory Animal Welfare guidelines. All animal experiments were approved by the Institutional Animal Care and Use Committee, Cairo University, Egypt (CU-IACUC) (code: CU-IACUC-II-F-9–16). The animal experiments were carried out after the owner's permission. Written informed consent was provided by each cat owner for the treatments. Treatment was applied with a high standard of veterinary care.

## Results

### Treatment

Animals’ responses to different treatments were described in supplementary data 1 and 2 which show the full treatment follow-up data of all cases in groups (TP, TS, and TSP) before and after the treatments and their figures.

### Checkerboard titration

The Laser-gene software analysis results were 76.65%, 60%, 88.8%, and 99.22% for TP53, MUC-1, MYC, and PCNA respectively. The optimal antigen concentration was (50 ng/well) and the optimum serum dilution was (1/160) which was applied in subsequent ELISA tests.

### The specificity and sensitivity of the AAbs to mammary tumors

**Figure 1:**
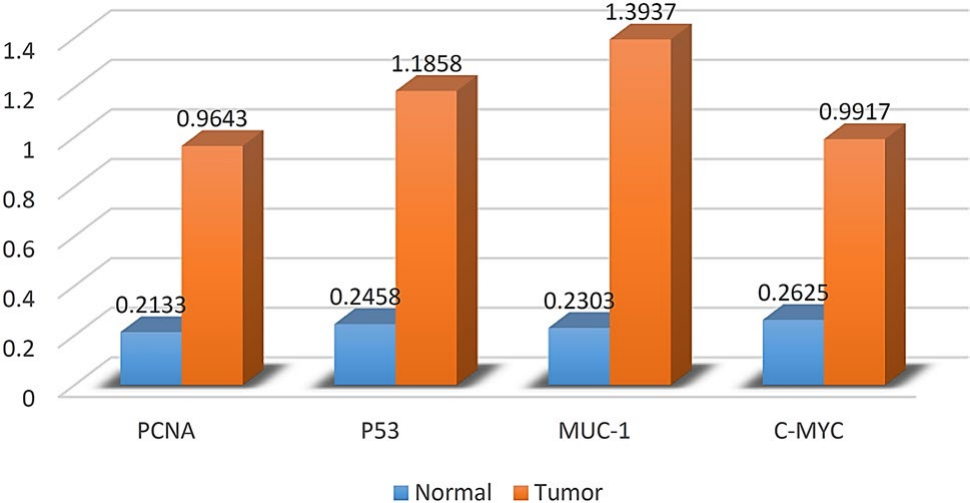
AAbs mean values measured for normal cats (n = 6, blue) and cats diagnosed with mammary tumor (n = 32, orange).

Results of ROC analysis (Table 2) showed that values of any of the four studied diagnostic markers before application of any treatment could significantly differentiate between the normal group (H; *n* = 6) and the cats diagnosed with mammary tumor (TN + TP + TS + TSP; *n* = 32) (Fig. 1).

**Table 2:** Mean ± standard error (*SE*) of AAbs values measured for normal cats (*n* = 6) and cats diagnosed with mammary tumor (*n* = 32), the area under the receiver operating characteristic curve (AUC) ± *SE* for the four studied AAbs and their 95% confidence intervals (C.I.), and *p*-value.

Variable	Markers			
	PCNA	P53	MUC-1	C-MYC
**Mean ± *SE***				
Normal	0.2133 ± 0.02	0.2458 ± 0.01	0.2303 ± 0.01	0.2625 ± 0.01
With tumor	0.9643 ± 0.08	1.1858 ± 0.09	1.3937 ± 0.10	0.9917 ± 0.05
**AUC**				
Estimate ± *SE*	0.9583 ± 0.03	0.9740 ± 0.03	0.9688 ± 0.03	1.00 ± 0.00
95% C.I	0.8931–1.024	0.9219–1.026	0.9075–1.030	1.000–1.000
*p*-value	0.0004	0.0003	0.0003	0.0001

**Figure 2:**
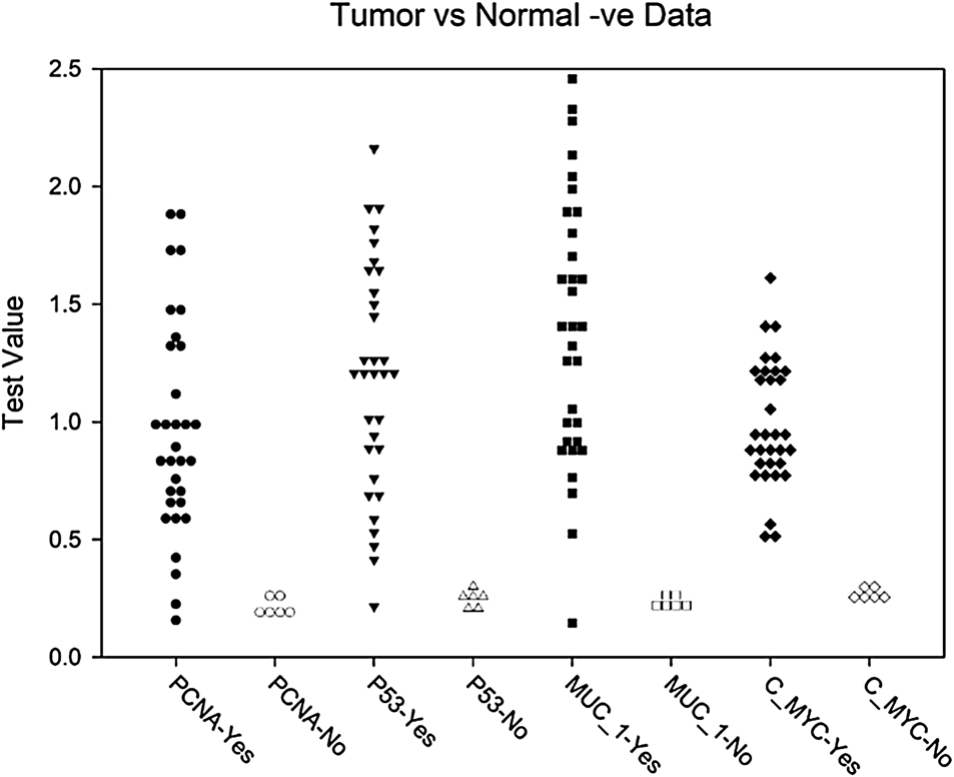
Measured values of four studied AAbs for six normal cats (No; white-filled symbols) and 32 cats diagnosed with mammary tumor (Yes; black-filled symbols) before application of any treatment. Figure was prepared using SigmaPlot v14.0 (Systat Software, San Jose, CA, USA).

As shown in Fig. 2, all 34 cats revealed positive ELISA for C-MYC, 33 cats were positive for MUC-1, and 32 cats were positive for PCNA and P53.

**Figure 3:**
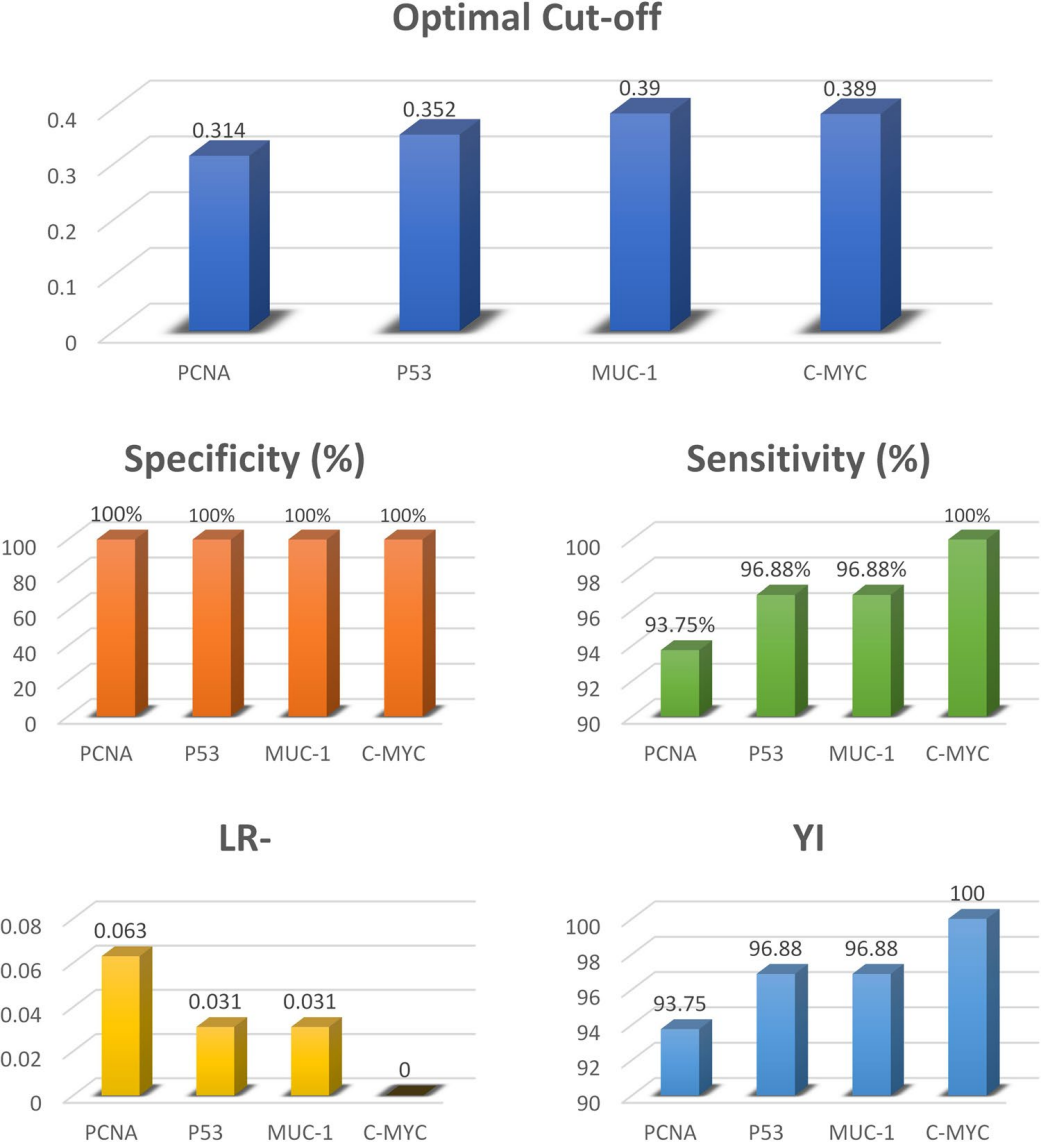
Optimal cut-off values of the four studied AAbs and their corresponding for sensitivity (%), specificity (%), negative likelihood ratio (LR-), and Youden’s index (YI).

**Figure 4:**
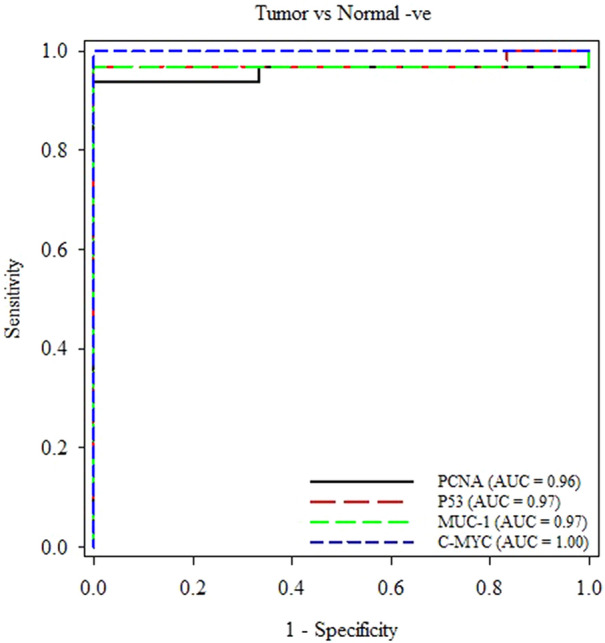
Comparison of receiver operating characteristic (ROC) curves of four different AAbs in mammary tumor detection. Figure was prepared using SigmaPlot v14.0 (Systat Software, San Jose, CA, USA).

Areas under the ROC curves were not different among the four studied AAbs (*p* > 0.05). The highest YI values indicated the optimal cut-off values for the studied AAbs with the corresponding specificity of 100% and sensitivity above 93.7% (Table 3) (Fig. 3). The results shown in Fig. 4 indicated the high specificity and sensitivity of each TAA in our panel for detection of AAbs using ELISA.

**Table 3:** Optimal cut-off values of the four studied AAbs and their corresponding for sensitivity (%), specificity (%), negative likelihood ratio (LR-), and Youden’s index (YI).

Marker	Optimal cut-off	Specificity (%)		Sensitivity (%)		LR-	YI
		Estimate	95% C.I	Estimate	95% C.I		
PCNA	0.314	100	54.07–100	93.75	79.19–99.23	0.063	93.75
P53	0.352	100	54.07–100	96.88	83.79–99.92	0.031	96.88
MUC-1	0.390	100	54.07–100	96.88	83.79–99.92	0.031	96.88
C-MYC	0.389	100	54.07–100	100	89.11–100	0.0	100.0

No positive likelihood ratio (LR +) values because all specificity values were 100%.

95%C.I. = 95% confidence intervals.

### The effect of different treatments on AAbs level

**Figure 5:**
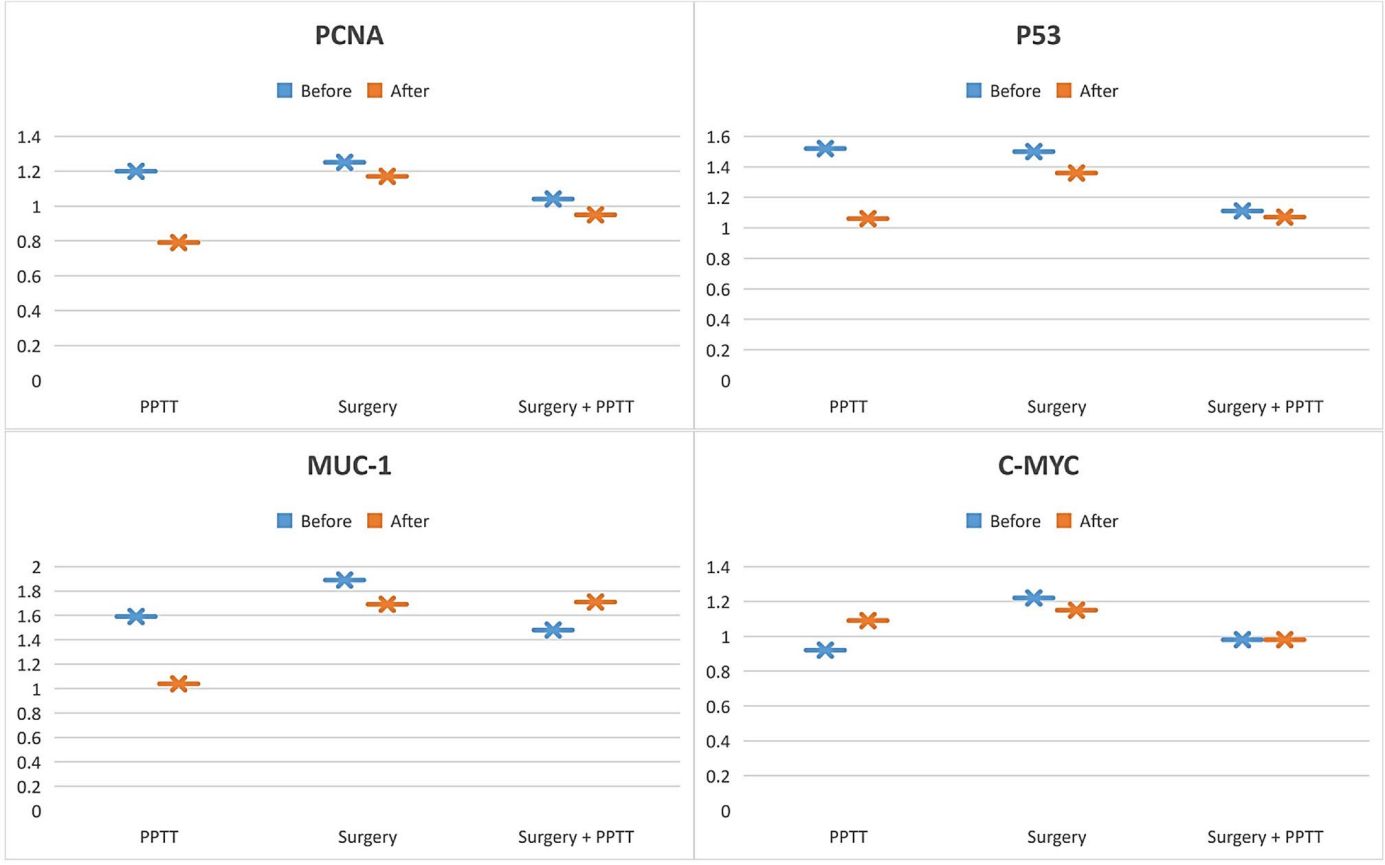
Comparison between AAbs values measured before treatment (first) and after treatment (last).

The efficiency of studied markers in monitoring treatment response before, during, and after treatment was evaluated. Values of PCNA, P53, and MUC-1 significantly decreased after treatment of mammary tumor using PPTT alone (*p* = 0.0078, 0.0022, and 0.0325 respectively) while no significant changes in their values in surgery only or combination therapy (Table 4) (Fig. 5).

**Table 4:** Comparison between AAbs values measured before treatment (first) and after treatment (last).

Marker	Sample	PPTT		Surgery		Surgery + PPTT	
		Mean ± *SE*	*t*_(df)_, *p*	Mean ± *SE*	*t*_(df)_, *p*	Mean ± *SE*	*t*_(df)_, *p*
PCNA	First	1.20 ± 014	*t*_(14)_ = 4.29 *p* = **0.0078**	1.25 ± 0.29	*t*_(14)_ = 0.49 *p* = 0.9959	1.04 ± 0.29	*t*_(14)_ = 0.71 *p* = 0.9776
	Last	0.79 ± 0.11		1.17 ± 0.21		0.95 ± 0.26	
P53	First	1.52 ± 0.13	*t*_(14)_ = 4.98 *p* = **0.0022**	1.50 ± 0.29	*t*_(14)_ = 0.85 *p* = 0.9522	1.11 ± 0.23	*t*_(14)_ = 0.31 *p* = 0.9995
	Last	1.06 ± 0.14		1.36 ± 0.25		1.07 ± 0.20	
MUC-1	First	1.59 ± 0.15	*t*_(14)_ = 3.52 *p* = **0.0325**	1.89 ± 0.33	*t*_(14)_ = 0.73 *p* = 0.9753	1.48 ± 0.26	*t*_(14)_ = 1.11 *p* = 0.8702
	Last	1.04 ± 0.17		1.69 ± 0.31		1.71 ± 0.25	
C-MYC	First	0.92 ± 0.07	*t*_(14)_ = 1.99 *p* = 0.3960	1.22 ± 0.25	*t*_(14)_ = 0.44 *p* = 0.9975	0.98 ± 0.16	*t*_(14)_ = 0.06 *p* = 0.9529
	Last	1.09 ± 0.07		1.15 ± 0.14		0.98 ± 0.09	

Significant results at *p* < 0.05 if (bold).

### The relationship between AAbs level and total tumor area

At any marker reading, regardless treatment group, total tumors area in any given cat had no significant effect on the corresponding PCNA (*F*_1,58_ = 0.60, *p* = 0.4407), P53 (*F*_1,57_ = 0.07, *p* = 0.7877), MUC-1 (*F*_1,61_ = 0.37, *p* = 0.5468), and C-MYC (*F*_1,58_ = 0.25, *p* = 0.6201) values. After controlling for total tumors area, there were no significant differences among treatments in all measured values of PCNA (*F*_2,28_ = 0.07, *p* = 0.9362), P53 (*F*_2,22_ = 3.13, *p* = 0.0632), MUC-1 (F2,29 = 1.04, *p* = 0.3673), and C-MYC (*F*_2,23_ = 1.01, *p* = 0.3790).

### The overall prevalence rate (PR)

The overall prevalence rate (PR) of developing **metastasis** after starting the treatment course was 17.7%. This PR was 0.0% in TS, 12.5% in TP, and 33.3% in TSP. The overall PR of the appearance of the **secondary tumor(s)** after starting the course of treatment was 35.3%. This PR was 0.0% in TS, 50.0% in TP, and 33.3% in TSP. The overall PR of the appearance of the **recurrent tumor(s)** after starting the course of treatment was 35.3%. This PR was 66.7% in TS, 25.0% in TP, and 33.3% in TSP.

### The efficiency of studied AAbs panel in early diagnosis

The efficiency of studied markers in the early diagnosis of **secondary tumors** and metastasis during the PPTT treatment course was evaluated. Within a month before the clinical appearance of secondary tumors, there was an increase in C-MYC values (Pre-predictive value *n* = 10, *Mean* ± *SE* = 0.86 ± 0.05 & predictive value *n* = 12 *Mean* ± *SE* = 0.90 ± 0.06). However, this increase was not statistically significant (*F*_1,8_ = 1.11, *p* = 0.3225). Also, the increases in “predictive” values of studied markers did not appear to be statistically significant (Table 5).

**Table 5:** Comparison between AAbs values measured just before the appearance of secondary tumors and metastasis (predictive), and last values measured before the predictive values (pre-predictive).

Event	Marker	Sampling time	Overall			PPTT			Surgery + PPTT (*n* = 2)	
			*N*	Mean ± *SE*	*F*_df_, *p*	*n*	Mean ± *SE*	*t*_(df)_ , *p*	Mean ± *SE*	*t*_(df)_ , *p*
Secondary tumor	PCNA	Pre-predictive	10	1.16 ± 0.13	*F*_1,11_ = 1.01 *p* = 0.3338	8	1.18 ± 0.11	*t*_(12)_ = 0.33 *p* = 0.9864	1.08 ± 0.60	*t*_(11)_ = 1.28 *p* = 0.5927
		Predictive	12	1.16 ± 0.13		10	1.13 ± 0.10		1.29 ± 0.73	
	P53	Pre-predictive	10	1.26 ± 0.13	*F*_1,11_ = 0.11 *p* = 0.7420	8	1.38 ± 0.12	*t*_(12)_ = 0.14 *p* = 0.9990	0.78 ± 0.09	*t*_(11)_ = 0.31 *p* = 0.9894
		Predictive	12	1.24 ± 0.12		10	1.34 ± 0.12		0.73 ± 0.11	
	MUC-1	Pre-predictive	10	1.52 ± 0.12	*F*_1,11_ = 0.07 *p* = 0.7903	8	1.53 ± 0.07	*t*_(12)_ = 0.01 *p* = 0.9908	1.52 ± 0.72	*t*_(11)_ = 0.31 *p* = 0.9892
		Predictive	12	1.48 ± 0.12		10	1.48 ± 0.11		1.46 ± 0.61	
	C-MYC	Pre-predictive	10	0.86 ± 0.05	*F*_1,8_ = 1.11 *p* = 0.3225	8	0.85 ± 0.06	*t*_(8)_ = 0.87 *p* = 0.8196	0.91 ± 0.05	*t*_(7)_ = 0.74 *p* = 0.8775
		Predictive	12	0.90 ± 0.06		10	0.89 ± 0.07		0.98 ± 0.05	
Metastasis	PCNA	Pre-predictive	5	1.20 ± 0.24	*F*_1,6_ = 0.04 *p* = 0.8540	3	1.29 ± 0.23	*t*_(6)_ = 0.09 *p* = 0.9997	1.08 ± 0.60	*t*_(6)_ = 0.32 *p* = 0.9873
		Predictive	5	1.26 ± 0.28		3	1.24 ± 0.30		1.29 ± 0.73	
	P53	Pre-predictive	5	1.12 ± 0.18	*F*_1,6_ = 0.21 *p* = 0.6628	3	1.34 ± 0.20	*t*_(6)_ = 0.89 *p* = 0.8111	0.78 ± 0.09	*t*_(6)_ = 0.13 *p* = 0.9990
		Predictive	5	1.26 ± 0.27		3	1.61 ± 0.29		0.73 ± 0.11	
	MUC-1	Pre-predictive	5	1.60 ± 0.31	*F*_1,6_ = 0.01 *p* = 0.9913	3	1.66 ± 0.37	*t*_(6)_ = 0.07 *p* = 0.9998	1.52 ± 0.72	*t*_(6)_ = 0.07 *p* = 0.9998
		Predictive	5	1.61 ± 0.32		3	1.71 ± 0.44		1.46 ± 0.61	
	C-MYC	Pre-predictive	5	0.87 ± 0.06	*F*_1,6_ = 3.12 *p* = 0.1278	3	0.84 ± 0.11	*t*_(6)_ = 2.08 *p* = 0.2596	0.91 ± 0.05	*t*_(6)_ = 0.58 *p* = 0.9340
		Predictive	5	1.02 ± 0.04		3	1.05 ± 0.05		0.98 ± 0.05	

The overall values were calculated regardless of treatments. Significant results at *p* < 0.05 if (bold).

The efficiency of studied markers in the early diagnosis of **recurrent tumors** after starting the course of treatment was evaluated. Although there were increases in some AAbs values within a month before the clinical appearance of recurrent tumors, however, the increases were not statistically significant (Table 6).

**Table 6:** Comparison between AAbs values measured just before the appearance of recurrent tumors (predictive), and last values measured before the predictive values (pre-predictive).

Marker	Sampling time	Overall (*n* = 6)		Surgery (*n* = 2)		PPTT (*n* = 3)		Surgery + PPTT (*n* = 1)	
		Mean ± *SE*	*F*_df_, *p*	Mean ± *SE*	*t*_(df)_ , *p*	Mean ± *SE*	*t*_(df)_ , *p*	Mean ± *SE*	*t*_(df)_ , *p*
PCNA	Pre-predictive	1.23 ± 0.18	*F*_1,3_ = 2.40 *p* = 0.2192	0.81 ± 0.24	*t*_(3)_ = 3.23 *p* = 0.1777	1.36 ± 0.18	*t*_(3)_ = 2.70 *p* = 0.2703	1.68 ± 0.0	*t*_(3)_ = 1.37 *p* = 0.7437
	Predictive	1.28 ± 0.22		1.37 ± 0.03		0.97 ± 0.31		2.02 ± 0.0	
P53	Pre-predictive	1.01 ± 0.09	*F*_1,3_ = 0.41 *p* = 0.5687	1.05 ± 0.06	*t*_(3)_ = 1.1 *p* = 0.8537	1.09 ± 0.13	*t*_(3)_ = 0.35 *p* = 0.9986	0.69 ± 0.0	*t*_(3)_ = 0.13 *p* = 0.9069
	Predictive	1.21 ± 0.23		1.51 ± 0.34		1.21 ± 0.36		0.61 ± 0.0	
MUC-1	Pre-predictive	1.74 ± 0.14	*F*_1,3_ = 0.08 *p* = 0.7905	1.53 ± 0.02	*t*_(3)_ = 1.28 *p* = 0.7848	1.72 ± 0.21	*t*_(3)_ = 1.65 *p* = 0.6199	2.24 ± 0.0	*t*_(3)_ = 0.35 *p* = 0.9987
	Predictive	1.63 ± 0.29		1.98 ± 0.02		1.25 ± 0.50		2.07 ± 0.0	
C-MYC	Pre-predictive	0.96 ± 0.04	*F*_1,3_ = 4.66 *p* = 0.1198	0.99 ± 0.05	*t*_(3)_ = 2.33 *p* = 0.3638	0.94 ± 0.07	*t*_(3)_ = 2.44 *p* = 0.3317	0.96 ± 0.0	*t*_(3)_ = 0.14 *p* = 0.8989
	Predictive	1.17 ± 0.06		1.27 ± 0.14		1.18 ± 0.03		0.93 ± 0.0	

The overall values were calculated regardless of treatments. Significant results at *p* < 0.05 if (bold).

**Figure 6:**
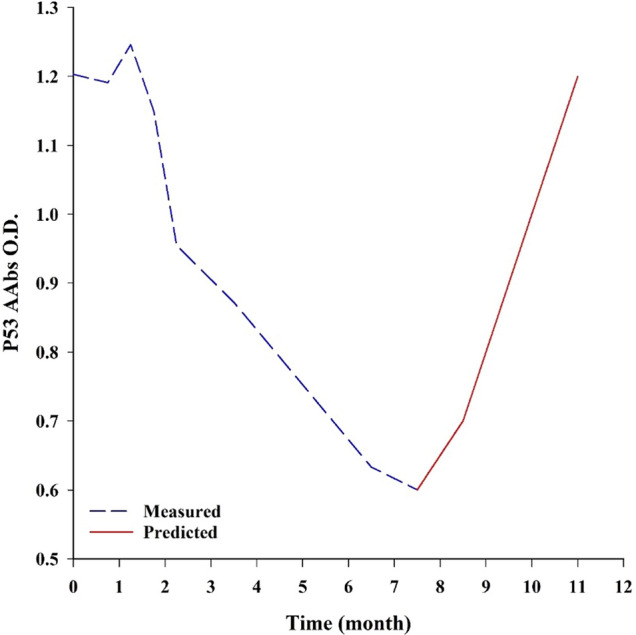
Measured and predicted values of P53 in a cat with a mammary tumor. The intersection between measured and predicted values marks the end of the PPTT treatment course. Figure was prepared using SigmaPlot v14.0 (Systat Software, San Jose, CA, USA).

The efficiency of studied markers in the early diagnosis of **primary tumors** was evaluated. Five apparently healthy (H; *n* = 5) cats showed positive AAbs values of 1.11 ± 0.10 for PCNA, 1.08 ± 0.12 for P53, 1.15 ± 0.10 for MUC-1, and 1.04 ± 0.07 for C-MYC. By following two of them for 11 months, they did not show any clinical manifestation of mammary carcinoma. Therefore, we could not validate AAbs’ efficiency in the early diagnosis of feline mammary carcinoma as in human breast cancer (Fig. 6).

### The median survival time

**Figure 7:**
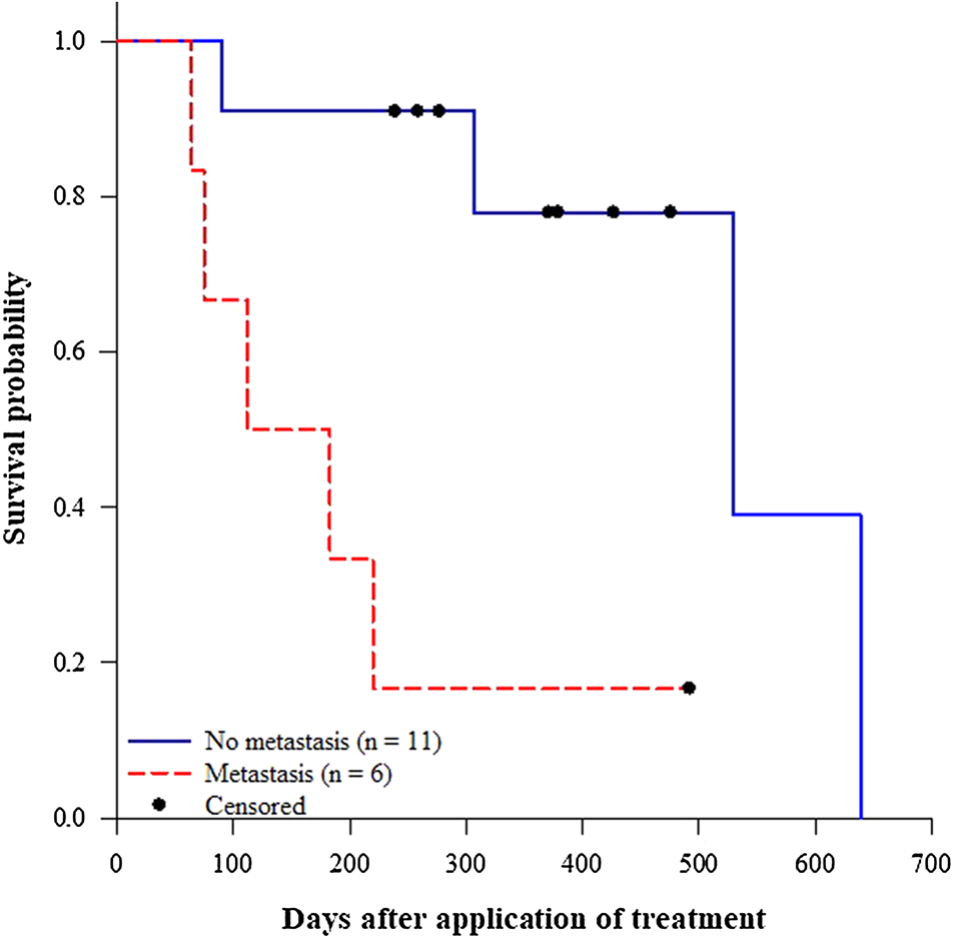
Effect of metastasis on survival probabilities of cats with mammary tumors (Log-rank test: *χ*^2^_(1)_ = 8.38, *p* = 0.004**)**. Figure was prepared using SigmaPlot v14.0 (Systat Software, San Jose, CA, USA).

**Figure 8:**
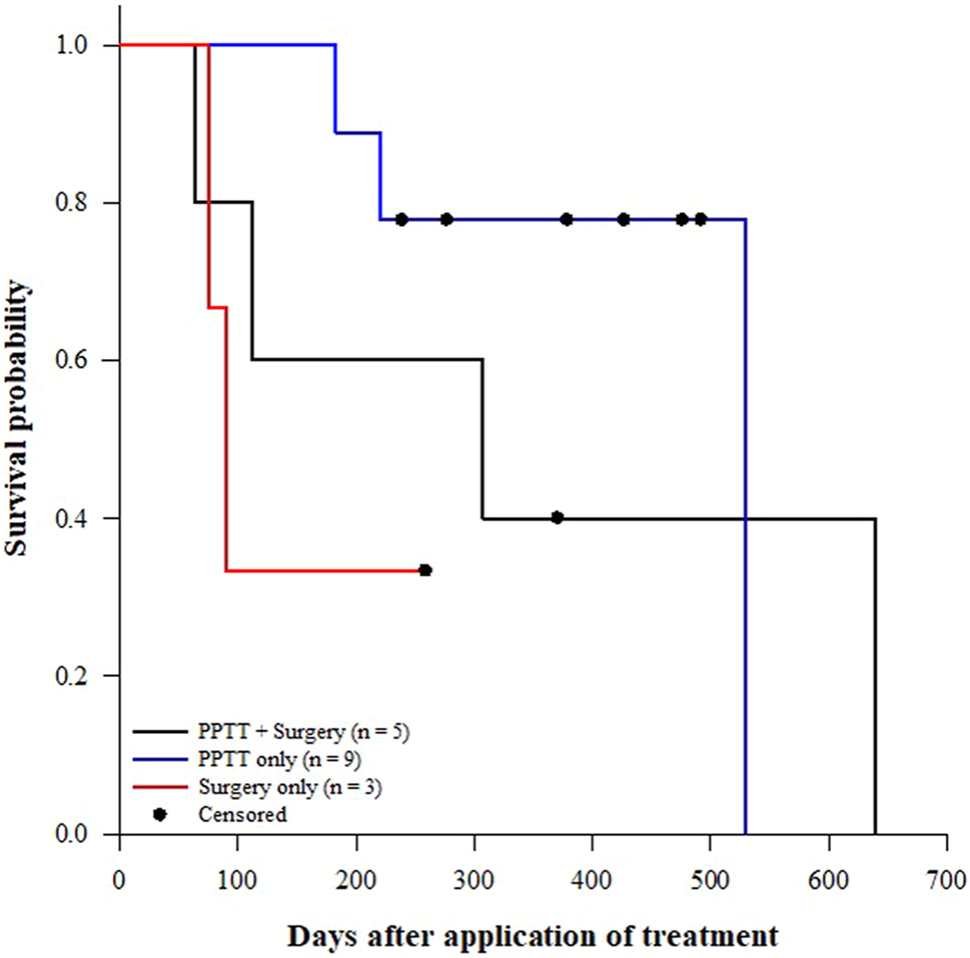
Effect of treatment on survival probabilities of cats with mammary tumors (Log-rank test: *χ*^2^_(2)_ = 3.13, *p* = 0.209). Figure was prepared using SigmaPlot v14.0 (Systat Software, San Jose, CA, USA).

In our study, across all treatments, the median survival time ± *SE* for cats without metastasis (530.0 ± 162.79 d; *n* = 11; 64% censored) was significantly higher (Fig. 7; Log-rank test: *χ*^2^_(1)_ = 8.38, *p* = 0.004) than that for cats with metastasis (112.0 ± 66.14 d; *n* = 6; 17% censored). While the median survival times ± *SE* for TS (90.0 ± 12.25 d; *n* = 3; 33% censored), TSP (307 ± 213.61 d; *n* = 5; 20.0% censored), and TP (530 ± 0.0 d; *n* = 9; 67% censored) were not significantly different (Fig. 8; Log-rank test: *χ*^2^_(2)_ = 3.13, *p* = 0.209).

## Discussion

Until now no confirmed model of TAAs for detection of AAbs performed with a sensitivity that would be acceptable for use in screening of breast cancer. In the present investigation, we tailored a panel of four TAAs, three of which (P53, MUC-1, and C-MYC) are the top among all breast cancer-associated tumor antigens used for detection of autoantibodies in terms of the frequency studies in addition to their close structural and function in BC. While the fourth TAAs (PCNA), is the first time to study the relationship between its AAbs and mammary tumors concerning that PCNA TAAs were detected in sera of diseased animals with various types of cancers such as duodenal, and in pulmonary cancers and considered as an important prognostic indicator of cancer. Authors added that PCNA TAA's high levels of expression were closely related to the occurrence, development, and prognosis of BC^37^. Although the detection of the autoantibodies accepted a raise of interest as a tool for diagnosis, early prediction, and monitoring of treatment response of different therapeutic modalities in cancers including breast cancer, PCNA autoantibodies have been seldom reported. In the present article, detection of PCNA AAbs in cats with mammary gland carcinoma revealed 93.75% sensitivity and 100% specificity confirmed the conclusions provided before about the usefulness of PCNA antibodies as a marker of BC and that these antibodies could serve as a highly effective detector of malignancy^38^.

While the negative control animal cut-off values for PCNA, P53, MUC-1, and C-MYC were 0.314, 0.351, 0.39, and 0.389, respectively. The obtained results indicated that the high specificity and sensitivity of each TAA in our panel for detection of AAbs using ELISA, however, a single TAA cannot be used alone as the single TAA can detect AAbs in other noncancerous diseases^39^.

Five of the 11 clinically normal cats showed positive levels of AAbs. These positive AAbs permit the suggestion that they were asymptomatically diseased animals (negative for clinical manifestation), but they are in the early stage of tumorigenesis and they are at the risk of mammary tumor appearance^8^. If so, our panel of TAA can be recommended for early diagnosis of BC, but this suggestion will be confirmed after a follow-up of these five cats until the appearance of primary mammary tumors as we follow them clinically for only 11 months. In women breast cancer, AAbs could be detected in sera of patients between seven and twenty-seven months before the cancer was diagnosed on the screening mammograms, and in a large cohort, AAbs could be detected up to four years before mammographic detection^40^. In the group treated with PPTT monotherapy, the AAbs panel values were non-significantly decreased during different cycles of PPTT. While the AAbs panel values after the end of treatment (after three months) were significantly decreased than the first values before initiation of PPTT therapy. We can indicate that the high level of AAbs before the treatment with PPTT correlated with tumor burden. After three months of the end of treatment, there was a significant decrease in AAbs level associated with a large reduction or complete disappearance of the tumor mass which means a decrease of the TAAs. The reduction of large tumor mass associated with eradication of TAAs hence the immune system would be exposed to fewer cancer antigens reducing the level of AAbs^41^. The group treated with PPTT monotherapy included two cases that were diagnosed with lung metastasis. One of them showed a significant decrease in AAbs values after three months from the end of local treatment of the mammary carcinoma with PPTT monotherapy and exhibited progression-free survival (PFS) and overall survival (OS) similar to the whole group cases. We can explain this by the abscopal phenomenon described before as a systemic anti-tumor immune response that reflects the regression of non-irradiated metastatic lesions at a distance from the primary site of irradiation which means a decrease tumor burden all over the body not only the local treated site^42^. Although there was a significant decrease in the AAbs panel values after the end of the treatment, these values did not reach values lower than the cut-off value (do not convert to normal value) which indicated AAbs persist in serum for long period. This data agrees with the previous study which recorded that patients whose serum is positive for P53 AAbs at diagnosis do not convert to negative even after the cancer is completely excised. It seems that once the patient’s immune system has been primed, there is sufficient P53 antigen available to maintain a long-term anti-p53 humoral response^43^. Depending on this data, we can suggest that any elevation of our AAbs panel values post decrease means the appearance of TAAs subsequent early tumorigenesis process before the appearance of clinical manifestation and paved us to suggest that this elevation can be used as an early prediction for secondary tumor, recurrence, or metastasis.

Also, in the group treated with PPTT monotherapy during the treatment course, there is a non-significance increase in AAbs level within a month before the clinical appearance of secondary tumors, recurrence, or metastasis. A previous study for the detection of P53 AAbs in colon cancer recorded that any temporal changes in the level of P53-AAbs could be closely correlated with disease progression or regression^44^. The reappearance of such antibodies may indicate a relapse of the disease. In the groups treated with surgery only or a combination between PPTT and surgery, there was no significant difference in AAbs panel values before and after the treatment with one month and a half (it is the available follow-up period). The persistence of AAbs after only one month and a half post-surgery confirmed that AAbs persist high for three months after the end of PPTT therapy. So, we can lay the non-significant changes in AAbs values in the TS and TSP groups on the short follow-up period which must be more than three months after the end of treatment. It was reported that AAbs persisted in the serum of colorectal cancer patients for at least six months after surgical removal of cancer^45^. This persistence of AAbs for three months after the end of PPTT therapy and after six months of surgical removal of cancer may be attributed to the long-lived population of memory plasma cells that can survive for months or years in circulation in a quiescent state^46^. The long overall survival of cats treated with PPTT monotherapy can be explained, as reported before^47^, by the abscopal effect of radiation therapy that does not only exert direct cytotoxic effects on tumor cells but also initiates immunogenic cell death causing production and release of the cytokines and chemokines into the tumor microenvironment. This causes chemoattraction and infiltration of dendritic cells (Dcs) to the site of the tumor. Activation of Dcs, which are essential antigen-presenting cells, up-regulation of cytotoxic T lymphocytes, induce a systemic antitumor response outside the irradiation field, and finally significantly delayed tumor growth and prolonged median survival time. But this abscopal effect caused by radiation monotherapy is quite rare and has not been extensively investigated.

## Conclusion

The AAbs profile detected by ELISA in sera of cats has distinct features reflecting a unique autoantibody repertoire. To enhance the sensitivity and specificity of ELISA detection of AAbs, we selected the most sensitive and specific tumor-associated BC antigens such as P53, MUC-1, PCNA, and C-MYC as coating antigens for ELISA plates. Our findings indicated that our tailored panel revealed high sensitivity and specificity in the diagnostic value of the four AAbs which may be used as potential biomarkers for the early detection of mammary carcinoma. Also, our tailored panel of AAbs can be used for tracking mammary carcinoma response to PPTT therapy.

## Supplementary Information

Supplementary Information 1.

Supplementary Information 2.

## Data Availability

All the relevant data about the study will be made available upon request.
