# Supplementary material for: The implication of autoantibodies in early diagnosis and monitoring of plasmonic photothermal therapy in the treatment of feline mammary carcinoma
Source: Sci Rep. 2021 May 17;11:10441. doi: 10.1038/s41598-021-89894-x (PMC8129074; doi:10.1038/s41598-021-89894-x)
Supplement: Supplementary file 1 — Supplementary Information 1. [file 41598_2021_89894_MOESM1_ESM.pdf]

The implication of autoantibodies in early diagnosis and monitoring of plasmonic photothermal therapy in the treatment of feline mammary carcinoma

Asmaa M. El-Rasikh [1], Haithem A. M. Farghali [2]*, Hisham A. Abdelrahman [3], Mostafa Elgaffary [4], Shaymaa Abdelmalek [1], Ibrahim A. Emam [2], Magdy A. Ghoneim [5], and Salah A. Selim [1]*

1 *Department of Microbiology, Immunology, and Mycology, Faculty of Veterinary Medicine, Cairo University, Giza, Egypt.*
2 *Department of Surgery, Anesthesiology, and Radiology, Faculty of Veterinary Medicine, Cairo University, Egypt.*
3 *Department of Veterinary Hygiene and management, Faculty of Veterinary Medicine, Cairo University, Egypt.*
4 *Department of Clinical Pathology, Faculty of Veterinary Medicine, Cairo University, Egypt.*
5 *Department of Biochemistry and molecular biology, Faculty of Veterinary Medicine, Cairo University, Egypt*

*Corresponding author: Salah A. Selim (Salah-Eldeen Abdelkerim Selim)
Affiliation: Professor of Microbiology, Immunology, and Mycology, Faculty of Veterinary Medicine, Cairo University, Egypt.
E-mail: dr.salahselim@hotmail.com
Address: Department of Microbiology, Immunology, and Mycology, Faculty of veterinary medicine, Cairo University, Giza, Egypt, Postal Code 12211.

*Corresponding author: Haithem A. M. Farghali (Haithem Ali Mohamed Ahmed Farghali)
Affiliation: Professor of surgery, anesthesiology and radiology, Faculty of veterinary medicine, Cairo University, Egypt.
E-mail: dr_haithem0@yahoo.com
Address: Department of surgery, anesthesiology and radiology, Faculty of veterinary medicine, Cairo University, Giza, Egypt, Postal Code 12211.

Asmaa M. El-Rasikh (Asmaa Mohammed Mohammed Mohammed El-Rasikh)
Affiliation: Master student, Department of Microbiology, Immunology, and Mycology, Faculty of Veterinary Medicine, Cairo University, Egypt.
E-mail: vet2014_sama@yahoo.com
Address: Department of Microbiology, Immunology, and Mycology, Faculty of Veterinary Medicine, Cairo University, Giza, Egypt, Postal Code 12211.

Hisham A. AbdelRahman (Hisham Ahmed AbdelRahman)
Affiliation: Lecturer of Veterinary Hygiene and Management, Faculty of Veterinary Medicine, Cairo University, Egypt.

E-mail: Hisham@auburn.edu
Address: Department of Veterinary Hygiene and Management, Faculty of Veterinary Medicine, Cairo University, Giza, Egypt, Postal Code 12211.

Mostafa ElGaffary (Mostafa Ahmed Mostafa Ali ElGaffary)
Affiliation: Lecturer of Veterinary Clinical Pathology, Faculty of Veterinary Medicine, Cairo University, Egypt.
E-mail: El.gaffary@cu.edu.eg
Address: Department of Clinical Pathology, Faculty of Veterinary Medicine, Cairo University, Giza, Egypt, Postal Code 12211.

Shaymaa Abdelmalek (Shaymaa AbdelMalek Mohammed AbdelHafez)
Affiliation: Lecturer of Microbiology, Immunology, and Mycology, Faculty of Veterinary Medicine, Cairo University, Egypt.
E-mail: shaymaa_malek@cu.edu.eg
Address: Department of Microbiology, Immunology, and Mycology, Faculty of Veterinary Medicine, Cairo University, Giza, Egypt, Postal Code 12211.

Ibrahim A. Emam (Ibrahim Abdallah Emam Ahmed Zeater)
Affiliation: Lecturer of Surgery, Anesthesiology and Radiology, Faculty of Veterinary Medicine, Cairo University, Egypt.
E-mail: dr.ibrahimabdallah2018@cu.edu.eg
Address: Department of Surgery, Anesthesiology and Radiology, Faculty of Veterinary Medicine, Cairo University, Giza, Egypt, Postal Code 12211.

Magdy A. Ghoneim (Magdy Ahmed Ghoneim)
Affiliation: Professor of Biochemistry and molecular biology, Faculty of Veterinary Medicine, Cairo University, Egypt.
E-mail: m2ghoneim@gmail.com
Address: Department of Biochemistry, Faculty of Veterinary Medicine, Cairo University, Giza, Egypt, Postal Code 12211.

Supplementary data (1)
The full treatment follow up data of all cases in groups TP, TS, and TSP

TP group case No.	cat name	cat age	sample date	week No.	tumor No.	tumor site	tumor size	PPTT session	% of size reduction	metastasis
1	Wala	15 Y	13/4/2019	0	1	L. caudal thoracic	1*1	0	0	0
				0	2	L. caudal abd	1*1	0	0	0
			5/5/2019	3	1	L. caudal thoracic	0.8*0.8	1	20	0
				3	2	L. caudal abd	0.8*0.8	1	20	0
			18/5/2019	5	1	L. caudal thoracic	0.6*0.6	2	40	0
				5	2	L. caudal abd	0.6*0.6	2	40	0
				5	3	L. inguinal	0.2*0.2	0	0	0
			1/6/2019	7	1	L. caudal thoracic	0.5*0.5	3	50	0
				7	2	L. caudal abd	0.5*0.5	3	50	0
				7	3	L. inguinal	0	1	100	0
					4	L. axillary L.N	0.5*0.5	0	0	
			15/6/2019	9	1	L. caudal thoracic	0.5*0.5	4	50	0
				9	2	L. caudal abd	0.5*0.5	4	50	0
				9	3	L. inguinal	0	1	100	0
				9	4	L. axillary L.N	0.5*0.5	1	0	0
			20/7/2019	14	1	L. caudal thoracic	0.5*0.5	5	50	0
				14	2	L. caudal abd	0	5	100	0
				14	3	L. inguinal	0	1	100	0
				14	4	L. axillary L.N	0.5*0.5	2	0	0

TP group case No.	cat name	cat age	sample date	week No.	tumor No.	tumor site	tumor size	PPTT session	% of size reduction	metastasis
2	Randa	10 Y	28/3/2019	0	1	L. caudal abd	2*1.5	0	0	1
			13/4/2019	2	1	L. caudal abd	1.5*0.8	1	60	1
				2	2	R. axillary L.N	0.2*0.2	0	0	1
			5/5/2019	5	1	L. caudal abd	1.3*1	2	60	1
				5	2	R. axillary L.N	0	1	100	1
			1/6/2019	9	1	L. caudal abd	1*1	3	66.7	1
				9	2	R. axillary L.N	0	2	100	1
				9	3	R. cranial thoracic	0.2*0.2	0	0	1
				9	4	R. caudal abd	0.2*0.2	0	0	1
			15/6/2019	11	1	L. caudal abd	1*1	4	66.7	1
				11	2	R. axillary L.N	0	2	100	1
				11	3	R. cranial thoracic	0.2*0.2	1	0	1
				11	4	R. caudal abd	0.2*0.2	1	0	1
			20/7/2019	16	1	L. caudal abd	0.7*0.7	5	83.7	1
				16	2	R. axillary L.N	0	2	100	1
				16	3	R. cranial thoracic	0.2*0.2	2	0	1
				16	4	R. caudal abd	0.2*0.2	2	0	1
			3/8/2019	18	1	L. caudal abd	0.5*0.7	6	88.3	1
				18	2	R. axillary L.N	0	2	100	1
				18	3	R. cranial thoracic	0	3	100	1
				18	4	R. caudal abd	0	3	100	1

TP group case No.	cat name	cat age	sample date	week No.	tumor No.	tumor site	tumor size	PPTT session	% of size reduction	metastasis
3	Tahaya	12 Y	1/6/2019	0	1	L. cranial thoracic	3*2	0	0	0
			15/6/2019	2	1	L. cranial thoracic	3*2	1	0	0
			20/7/2019	7	1	L. cranial thoracic	1*1	2	83.3	0
			3/8/2019	9	1	L. cranial thoracic	1*1	3	83.3	0
			23/11/2019	25	1	L. cranial thoracic	0	4	100	0

TP group case No.	cat name	cat age	sample date	week No.	tumor No.	tumor site	tumor size	PPTT session	% of size reduction	metastasis
4	Dalia	7 Y	6/12/2019	0	1	L. caudal thoracic	5*4	0	0	0
			20/12/2019	2	1	L. caudal thoracic	5*4	1	0	0
			3/1/2020	4	1	L. caudal thoracic	5*4	2	0	0
				4	2	R. cranial abd	5*4	0	0	0
			17/1/2020	6	1	L. caudal thoracic	5*4	3	0	0
				6	2	R. cranial abd	5*4	1	0	0
			14/2/2020	10	1	L. caudal thoracic	5*4	5	0	0
				10	2	R. cranial abd	5*4	3	0	0
			28/2/2020	12	1	L. caudal thoracic	5*4	6	0	0
				12	2	R. cranial abd	5*4	4	0	0
			13/3/2020	14	1	L. caudal thoracic	N. A	7	reduction	N. A
				14	2	R. cranial abd	N. A	5	reduction	N. A

TP group case No.	cat name	cat age	sample date	week No.	tumor No.	tumor site	tumor size	PPTT session	% of size reduction	metastasis
5	Sara	8 Y	2/3/2019	0	1	L. caudal thoracic	3*2.5	0	0	0
					2	R. caudal thoracic	2*1.5	0	0	0
			23/3/2019	3	1	L. caudal thoracic	3*1.7	1	32	0
				3	2	R. caudal thoracic	2*1	1	33.3	0
			6/4/2019	5	1	L. caudal thoracic	2.2*1	2	70.6	0
				5	2	R. caudal thoracic	1.1*1	2	36.7	0
			5/5/2019	8	1	L. caudal thoracic	2.5*1	3	66.7	N. A
				8	2	R. caudal thoracic	2*1	3	33.3	N. A
			18/5/2019	10	1	L. caudal thoracic	2.5*1.5	4	50	N. A
				10	2	R. caudal thoracic	2*1	4	33.3	N. A
				10	3	L. cranial thoracic	0.2*0.2	0	0	N. A
				10	4	L. cranial abd	0.5*0.5	0	0	N. A
				10	5	R. inguinal	1*0.5	0	0	N. A
			1/6/2019	14	1	L. caudal thoracic	2*1.4	5	62.7	1
				14	2	R. caudal thoracic	2*1.7	5	0	1
				14	3	L. cranial thoracic	0	1	100	1
				14	4	L. cranial abd	0.2*0.2	1	60	1
				14	5	R. inguinal	1*0.7	1	0	1
										metastasis
		death	9/2020							

TP group case No.	cat name	cat age	sample date	week No.	tumor No.	tumor site	tumor size	PPTT session	% of size reduction	metastasis
6	Dr/ Elfar	7 Y	10/9/2018	0	1	L. caudal thoracic	4.5*3	0	0	0
			6/10/2018	4	1	L. caudal thoracic	4.5*3	1	0	0
				4	2	R. inguinal	0.2*0.2	0	0	0
			20/10/2018	6	1	L. caudal thoracic	4.5*3	2	0	0
				6	2	R. inguinal	0	1	100	0
			12/1/2019	16	1	L. caudal thoracic	3.5*2.5	3	35.1	0
				16	2	R. inguinal	0	1	100	0
			9/10/2019	metastasis	N. A	N. A	N. A	4	N. A	1
			5/2020	death	N. A	N. A	N. A		N. A	1

TP group case No.	cat name	cat age	sample date	week No.	tumor No.	tumor site	tumor size	PPTT session	% of size reduction	metastasis
7	Tanta	10 Y	1/8/2018	0	1	L. caudal abd	0.2*0.2	0	0	0
				0	2	L. inguinal	1*1	0	0	0
				0	3	R. inguinal	1*1	0	0	0
			10/9/2018	6	1	L. caudal abd	0	1	100	0
				6	2	L. inguinal	1*1	1	0	0
				6	3	R. inguinal	1*1	1	0	0
			6/10/2018	10	1	L. caudal abd	0	1	100	0
				10	2	L. inguinal	1*1	2	0	0
				10	3	R. inguinal	1*1	2	0	0
			3/11/2018	14	1	L. caudal abd	0	1	100	0
				14	2	L. inguinal	0.5*0.5	3	50	0
				14	3	R. inguinal	3*2	3	0	0
				14	4	R. caudal abd	0.2*0.2	0	0	0
			13/1/2020	death		recurrence +metastasis	N. A		N. A	1

TP group case No.	cat name	cat age	sample date	week No.	tumor No.	tumor site	tumor size	PPTT session	% of size reduction	metastasis
8	Nayomi	13 Y	29/10/2019	0	1	L. cranial abd	0.5*0.5	0	0	0
				0	2	L. caudal abd	0.2*0.2	0	0	
				0	3	in-between	1*1	0	0	0
			15/11/2019	2	1	L. cranial abd	0	1	100	0
				2	2	L. caudal abd	0	1	100	0
				2	3	in-between	1*1	1	0	0
			29/11/2019	4	1	L. cranial abd	0	1	100	0
				4	2	L. caudal abd	0	1	100	0
				4	3	In-between	1*1	2	0	0
			13/12/2019	6	1	L. cranial abd	0	1	100	0
				6	2	L. caudal abd	0	1	100	0
				6	3	In-between	1*1	3	0	0
			27/12/2019	8	1	L. cranial abd	0	1	100	0
				8	2	L. caudal abd	0	1	100	0
				8	3	In-between	1*1	4	0	0
			10/1/2019	10	1	L. cranial abd	0	1	100	0
				10	2	L. caudal abd	0	1	100	0
				10	3	In-between	1*1	5	0	0
			7/2/2020	14	1	L. cranial abd	0	1	100	0
				14	2	L. caudal abd	0	1	100	0
				14	3	In-between	1*1	6	0	0

TP group case No.	cat name	cat age	sample date	week No.	tumor No.	tumor site	tumor size	PPTT session	% of size reduction	metastasis
9	Gebsi	18 Y	19/7/2019	0	1	L. inguinal	0.2*0.2	0	0	0
			29/10/2019	14	1	L. inguinal	0	1	100	0
			15/11/2019	16	1	L. inguinal	0	1	100	0
			13/12/2019	20	1	L. inguinal	0	1	100	0

TP group case No.	cat name	cat age	sample date	week No.	tumor No.	tumor site	tumor size	PPTT session	% of size reduction	metastasis
10	Yasmin	19 Y	20/7/2019	0	1	R. inguinal	2.5*1.5	0	0	1
				0	2	L. caudal abd	1*0.7	0	0	1
			3/8/2019	2	1	R. inguinal	2*1.5	1	20	1
				2	2	L. caudal abd	1*0.7	1	0	1
			25/2/2020	death						

TS group case No.	cat name	cat age	sample date	week No.	tumor No.	tumor site	tumor size	PPTT session	% of size reduction	metastasis
1	Nahed	15 Y	7/10/2019	0	1	R. axillary L.N	N. A	0	0	1
				0	2	R. cranial thoracic	N. A	0	0	1
				0	3	R. caudal thoracic	N. A	0	0	1
				0	4	R. cranial abd	N. A	0	0	1
			19/10/2019	2	1,2,3,4	0	0	0	100	1
			9/11/2019	5	1,2,3,4	0	0	0	100	1
			26/11/2019		recurrence	N. A	N. A	0	N. A	1
			21/12/2019		death					

TS group case No.	cat name	cat age	sample date	week No.	tumor No.	tumor site	tumor size	PPTT session	% of size reduction	metastasis
2	Kefaya	10 Y	9/11/2019	0	1	R. cranial abd	2.5*2.5	0	0	0
					2	R. caudal abd	2.5*2.5	0	0	0
			23/11/2019	2	1,2	0	0	0	100	0
			7/12/2019	4	1,2	0	0	0	100	0
			7/2/2020	death						

TS group case No.	cat name	cat age	sample date	week No.	tumor No.	tumor site	tumor size	PPTT session	% of size reduction	metastasis
3	Fatma	9 Y	16/11/2019	0	1	R. axillary L.N	1*1	0	0	0
				0	2	R. caudal thoracic	2*1.5	0	0	0
				0	3	R. cranial abd	2*1.5	0	0	0
			30/11/2019	2	1,2,3	0	0	0	100	0
			28/12/2019	6	1,2,3	0	0	0	100	0
			3/2020	N. A	recurrence	N. A	N. A	0	N. A	N. A

TSP group case No.	cat name	cat age	sample date	week number	tumor number	tumor site	tumor size	PPTT session	% of size reduction	metastasis
1	Dr/ Ahmed	7 Y	1/8/2018	0	1	L. cranial abd	5*5	0	0	0
					2	L. caudal abd	5*5	0	0	0
			14/8/2018	2	1,2	0	0	1	100	0
			28/8/2018	4	1,2	0	0	1	100	0
			10/9/2018	6	1,2	0	0	1	100	0
					3	L. caudal thoracic	1*1	0	0	0
			9/10/2019	metastasis						
			5/2020	death						

TSP group case No.	cat name	cat age	sample date	week No.	tumor No.	tumor site	tumor size	PPTT session	% of size reduction	metastasis
2	Aya	10 Y	13/4/2019	0	1	L. inguinal	3.5*1.5	4	0	0
					2	R. inguinal	3.5*1.5	6	0	0
			5/5/2019	3	1	0	0	5	100	0
				3	2	0	0	7	100	0
			15/6/2019	12	1	0	0	5	100	0
					2	0	0	7	100	0
			11/2019	recurrence	new	metastasis				
			1/2020	death						

TSP group case No.	cat name	cat age	sample date	week No.	tumor No.	tumor site	tumor size	PPTT session	% of size reduction	metastasis
3	Roba	14 Y	29/3/2019	0	5	all left line	N. A	0	0	1
				0	6	R. cranial abd	2.5*3.5	0	0	
			12/4/2019	2	5	0	0	1	100	1
				2	6	R. cranial abd	2.5*3.5	1	0	1
			3/5/2019	4	5	0	0	1	100	1
				4	6	R. cranial abd	3*3.5	2	0	1
			يونيو 2019	death						

TSP group case No.	cat name	cat age	sample date	week No.	tumor No.	tumor site	tumor size	PPTT session	% of size reduction	metastasis
4	Esraa	12 Y	25/10/2019	0	1	L. axillary L.N	4*3.5	0	0	1
					2	L. cranial thoracic	2*2	0	0	1
					3	L. caudal thoracic	2*2	0	0	1
			15/11/2019	3	1,2,3	0	0	1	100	1
			13/12/2019	7	1,2,3	0	0	1	100	1
			14/2/2020	death						

TSP group case No.	cat name	cat age	sample date	week No.	tumor No.	tumor site	tumor size	PPTT session	% of size reduction	metastasis
5	Meral	9 Y	17/10/2018	0	1	L. cranial abd	6*4	0	0	0
				0	2	L. inguinal	1*1	0	0	0
			3/11/2018	2	1	0	0	1	100	0
				2	2	L. inguinal	0.5*0.5	1	50	0
			20/8/2019	death						

TSP group case No.	cat name	cat age	sample date	week No.	tumor No.	tumor site	tumor size	PPTT session	% of size reduction	metastasis
6	Dr/ Nabil	10 Y	27/7/2019	0	1	R. cranial abd	5*4	0	0	0
				0	2	R. caudal abd	5*4	0	0	0
				0	3	R. inguinal	4*2	0	0	0
			3/8/2019	1	1,2,3	0	0	1	100	0
			12/2019	N. A	recurrence	N. A	N. A	N. A	N. A	N. A

(TP) tumor-PPTT alone, (TS) tumor-surgery alone, (TSP) tumor-surgery, and PPTT, (N. A) Not available, (L) left, (R) right, (abd) abdominal, (L. N) lymph node, (0) x-ray not show metastasis, (1) x-ray show metastasis.
