# Supplementary material for: The implication of autoantibodies in early diagnosis and monitoring of plasmonic photothermal therapy in the treatment of feline mammary carcinoma
Source: Sci Rep. 2021 May 17;11:10441. doi: 10.1038/s41598-021-89894-x (PMC8129074; doi:10.1038/s41598-021-89894-x)

The implication of autoantibodies in early diagnosis and monitoring of plasmonic photothermal therapy in the treatment of feline mammary carcinoma

Asmaa M. El-Rasikh [1], Haithem A. M. Farghali [2]*, Hisham A. Abdelrahman [3], Mostafa Elgaffary [4], Shaymaa Abdelmalek [1], Ibrahim A. Emam [2], Magdy A. Ghoneim [5], and Salah A. Selim [1]*

1 *Department of Microbiology, Immunology, and Mycology, Faculty of Veterinary Medicine, Cairo University, Giza, Egypt.*
2 *Department of Surgery, Anesthesiology, and Radiology, Faculty of Veterinary Medicine, Cairo University, Egypt.*
3 *Department of Veterinary Hygiene and management, Faculty of Veterinary Medicine, Cairo University, Egypt.*
4 *Department of Clinical Pathology, Faculty of Veterinary Medicine, Cairo University, Egypt.*
5 *Department of Biochemistry and molecular biology, Faculty of Veterinary Medicine, Cairo University, Egypt*

*Corresponding author: Salah A. Selim (Salah-Eldeen Abdelkerim Selim)
Affiliation: Professor of Microbiology, Immunology, and Mycology, Faculty of Veterinary Medicine, Cairo University, Egypt.
E-mail: dr.salahselim@hotmail.com
Address: Department of Microbiology, Immunology, and Mycology, Faculty of veterinary medicine, Cairo University, Giza, Egypt, Postal Code 12211.

*Corresponding author: Haithem A. M. Farghali (Haithem Ali Mohamed Ahmed Farghali)
Affiliation: Professor of surgery, anesthesiology and radiology, Faculty of veterinary medicine, Cairo University, Egypt.
E-mail: dr_haithem0@yahoo.com
Address: Department of surgery, anesthesiology and radiology, Faculty of veterinary medicine, Cairo University, Giza, Egypt, Postal Code 12211.

Asmaa M. El-Rasikh (Asmaa Mohammed Mohammed Mohammed El-Rasikh)
Affiliation: Master student, Department of Microbiology, Immunology, and Mycology, Faculty of Veterinary Medicine, Cairo University, Egypt.
E-mail: vet2014_sama@yahoo.com
Address: Department of Microbiology, Immunology, and Mycology, Faculty of Veterinary Medicine, Cairo University, Giza, Egypt, Postal Code 12211.

Hisham A. AbdelRahman (Hisham Ahmed AbdelRahman)
Affiliation: Lecturer of Veterinary Hygiene and Management, Faculty of Veterinary Medicine, Cairo University, Egypt.
E-mail: Hisham@auburn.edu
Address: Department of Veterinary Hygiene and Management, Faculty of Veterinary Medicine, Cairo University, Giza, Egypt, Postal Code 12211.

Mostafa ElGaffary (Mostafa Ahmed Mostafa Ali ElGaffary)

Affiliation: Lecturer of Veterinary Clinical Pathology, Faculty of Veterinary Medicine, Cairo University, Egypt.
E-mail: El.gaffary@cu.edu.eg
Address: Department of Clinical Pathology, Faculty of Veterinary Medicine, Cairo University, Giza, Egypt, Postal Code 12211.

Shaymaa Abdelmalek (Shaymaa AbdelMalek Mohammed AbdelHafez)
Affiliation: Lecturer of Microbiology, Immunology, and Mycology, Faculty of Veterinary Medicine, Cairo University, Egypt.
E-mail: shaymaa_malek@cu.edu.eg
Address: Department of Microbiology, Immunology, and Mycology, Faculty of Veterinary Medicine, Cairo University, Giza, Egypt, Postal Code 12211.

Ibrahim A. Emam (Ibrahim Abdallah Emam Ahmed Zeater)
Affiliation: Lecturer of Surgery, Anesthesiology and Radiology, Faculty of Veterinary Medicine, Cairo University, Egypt.
E-mail: dr.ibrahimabdallah2018@cu.edu.eg
Address: Department of Surgery, Anesthesiology and Radiology, Faculty of Veterinary Medicine, Cairo University, Giza, Egypt, Postal Code 12211.

Magdy A. Ghoneim (Magdy Ahmed Ghoneim)
Affiliation: Professor of Biochemistry and molecular biology, Faculty of Veterinary Medicine, Cairo University, Egypt.
E-mail: m2ghoneim@gmail.com
Address: Department of Biochemistry, Faculty of Veterinary Medicine, Cairo University, Giza, Egypt, Postal Code 12211.

Supplementary data (2)

Figures of some cases of TP, TS, and TSP groups

Figure 1. Case No. 2 in TP group before treatment

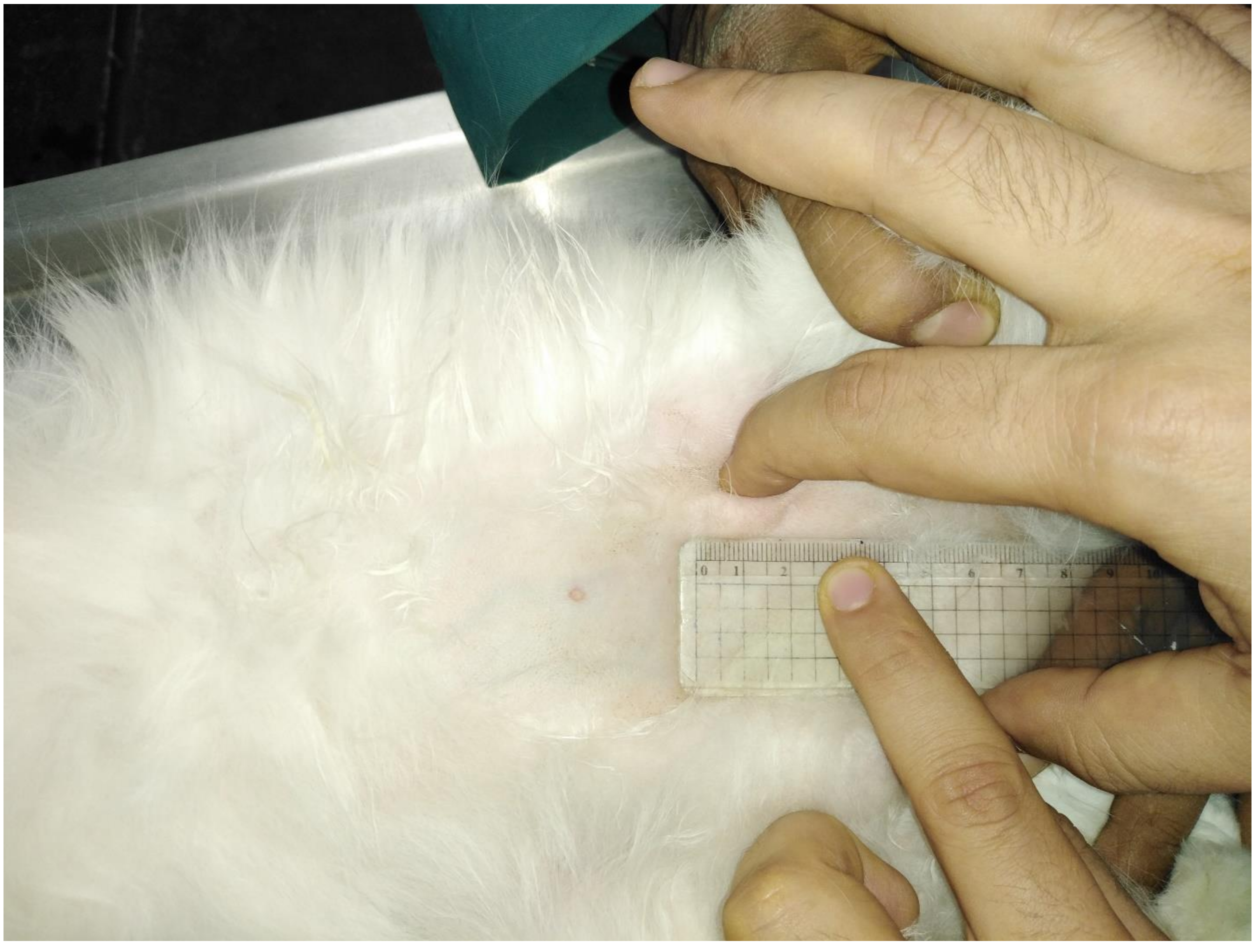

Figure 2. Case No. 2 of TP group after 6 PPTT sessions

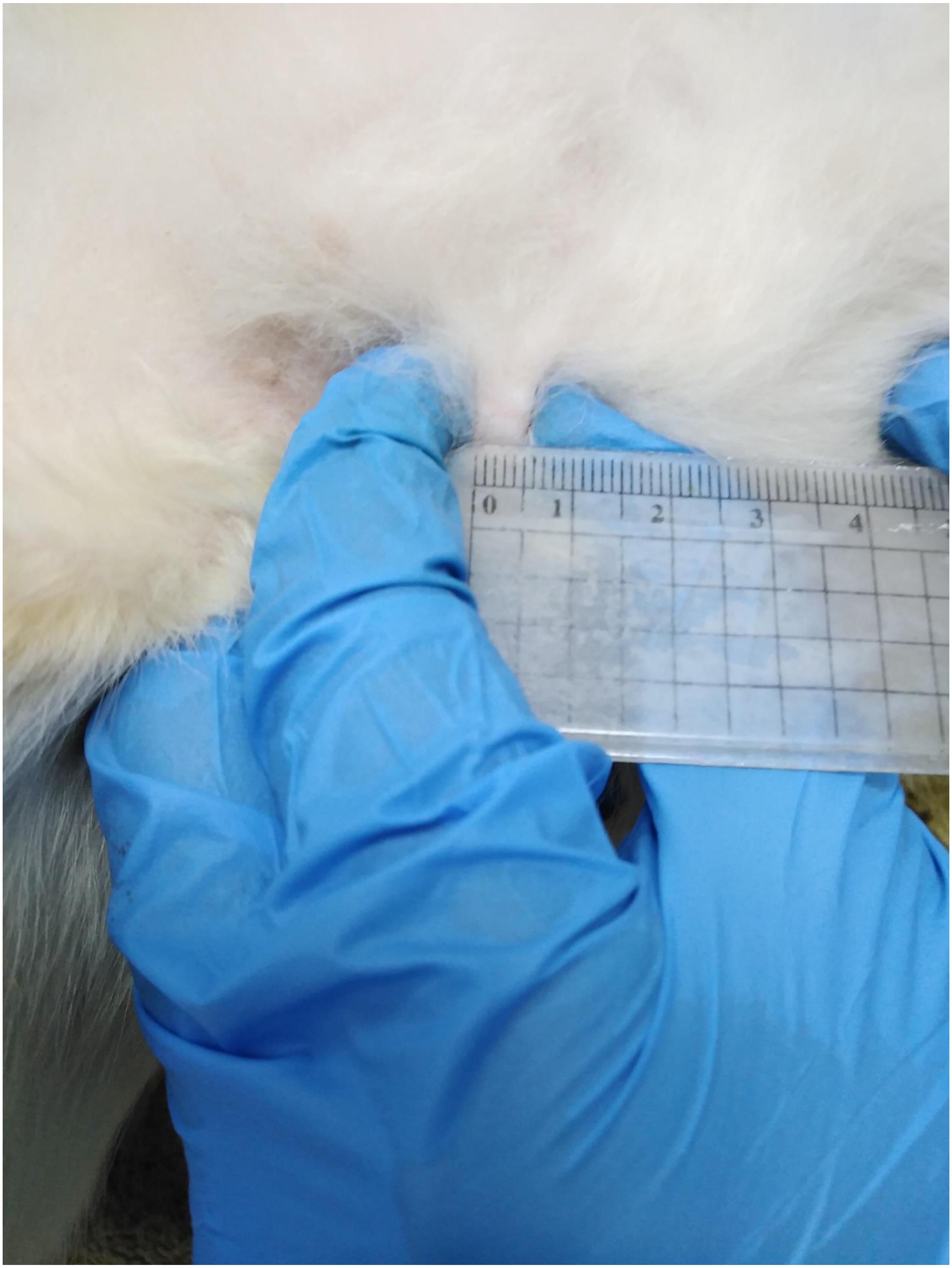

Figure 3. Case No. 3 of TP group before treatment

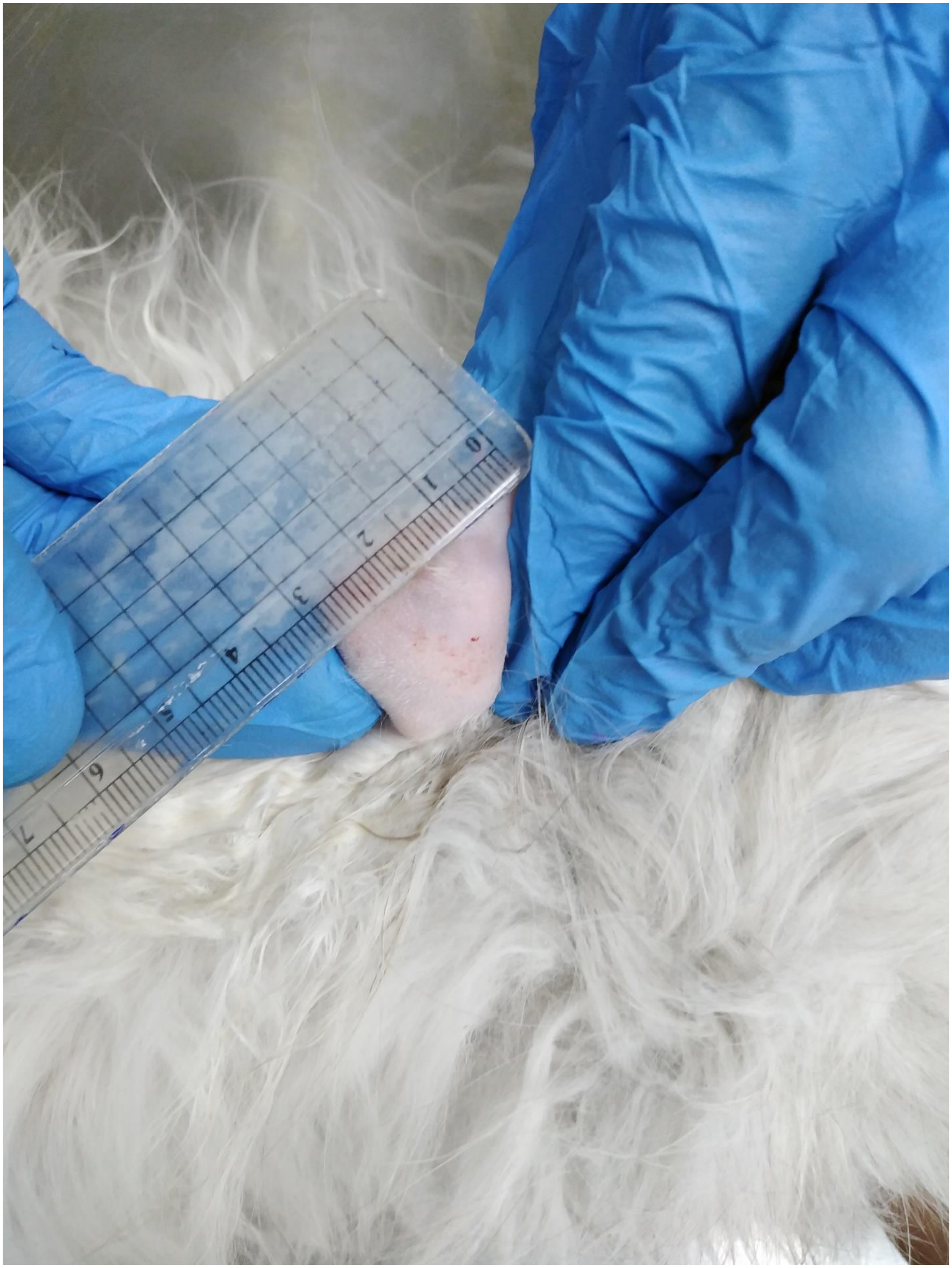

Figure 4. Case No. 3 of TP group after 2 sessions of PPTT

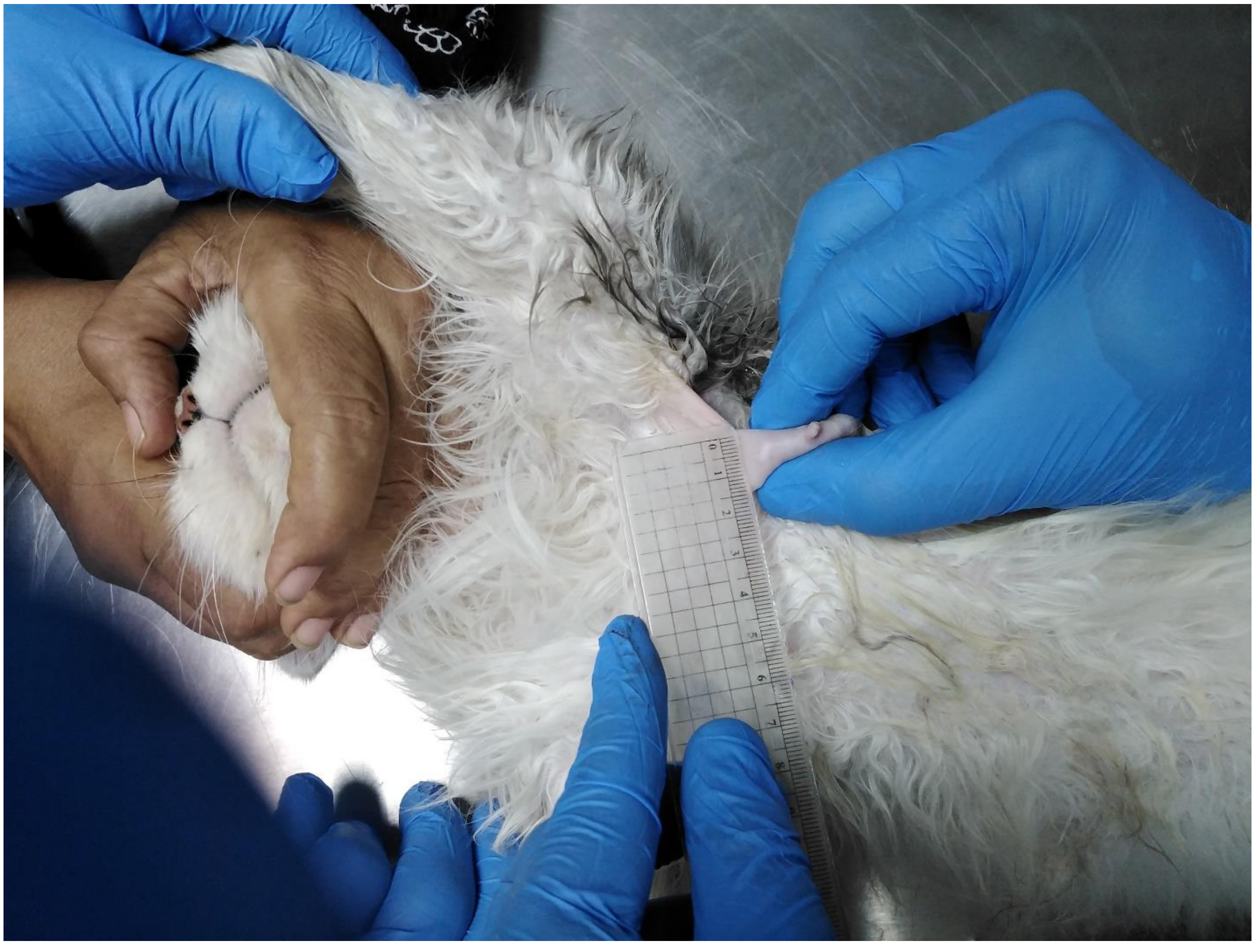

Figure 5. Case No. 4 of TP group before treatment

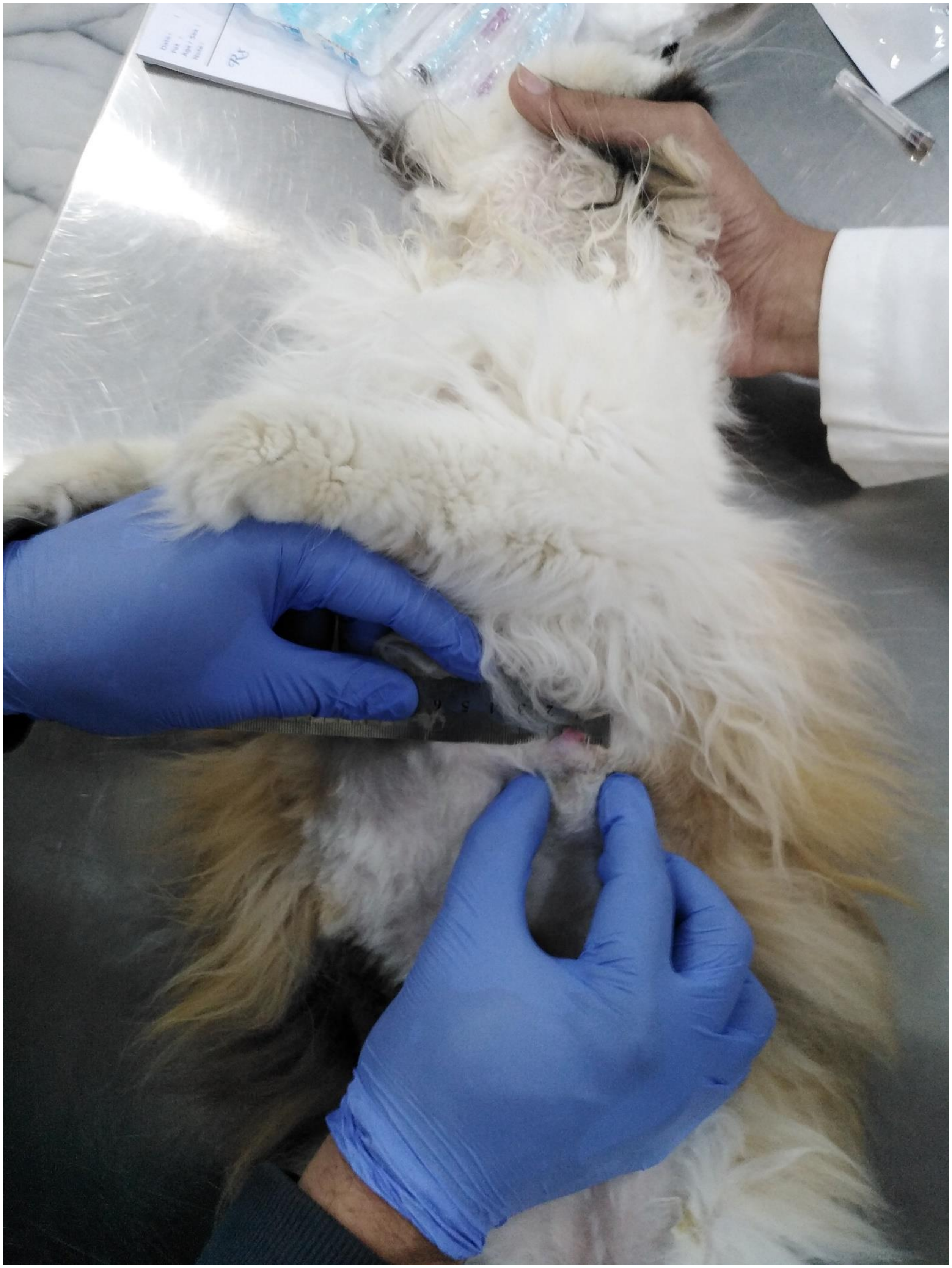

Figure 6. Case 4 of PPTT monotherapy group (after 6 PPTT sessions)

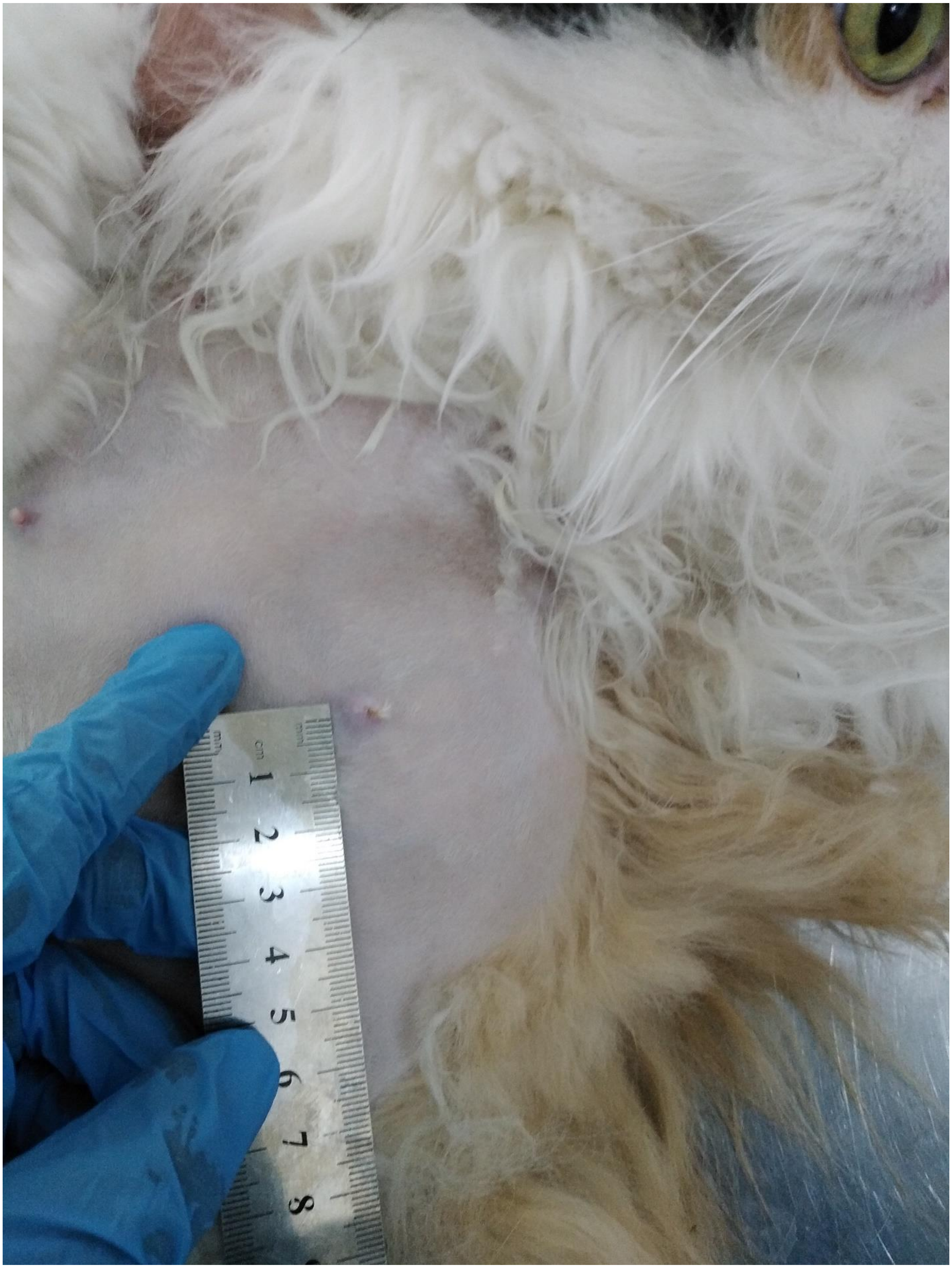

Figure 7. Case No. 2 of TS group before surgery

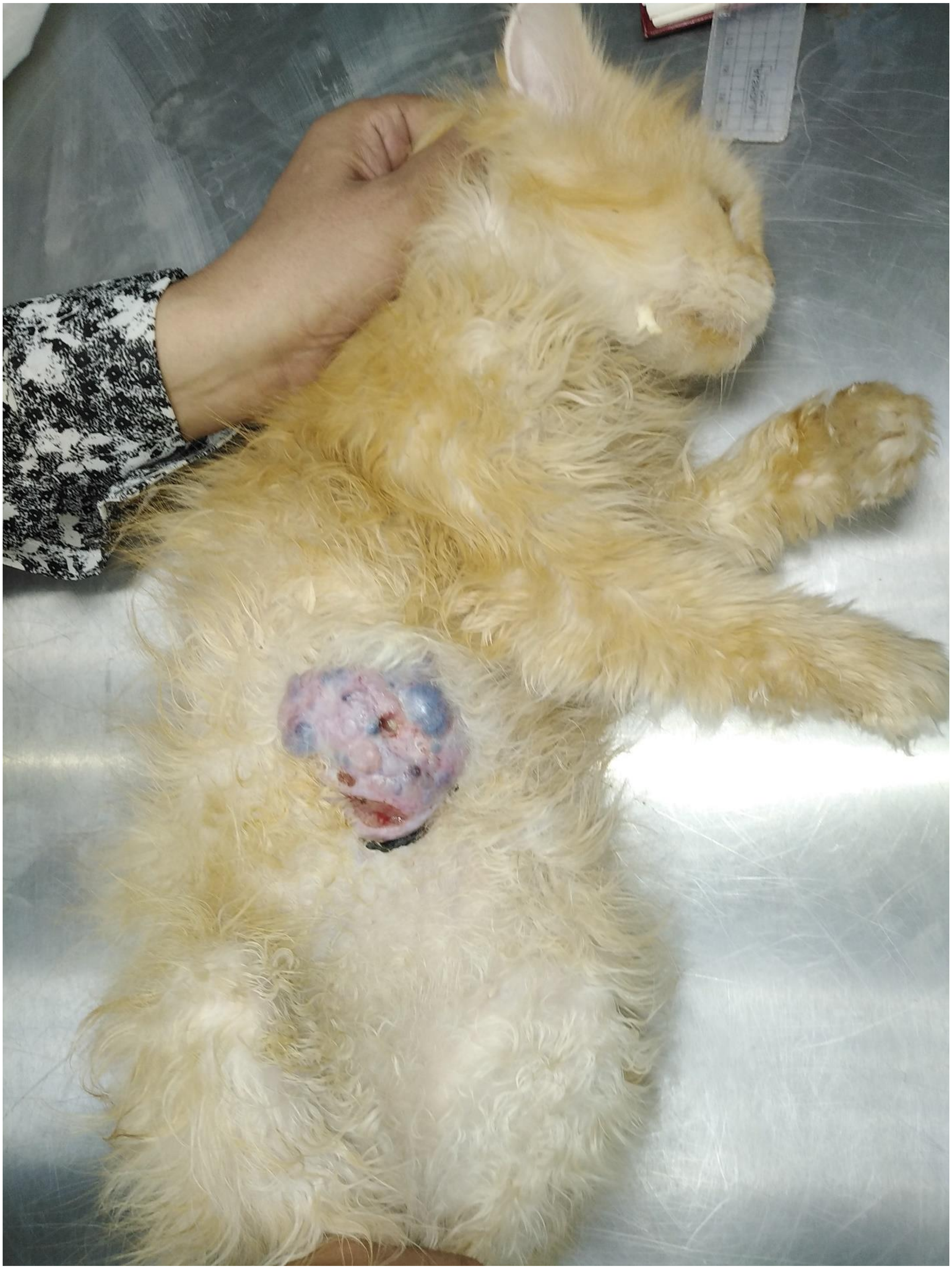

Figure 8. Case No. 2 of TS group after 2 weeks of surgery

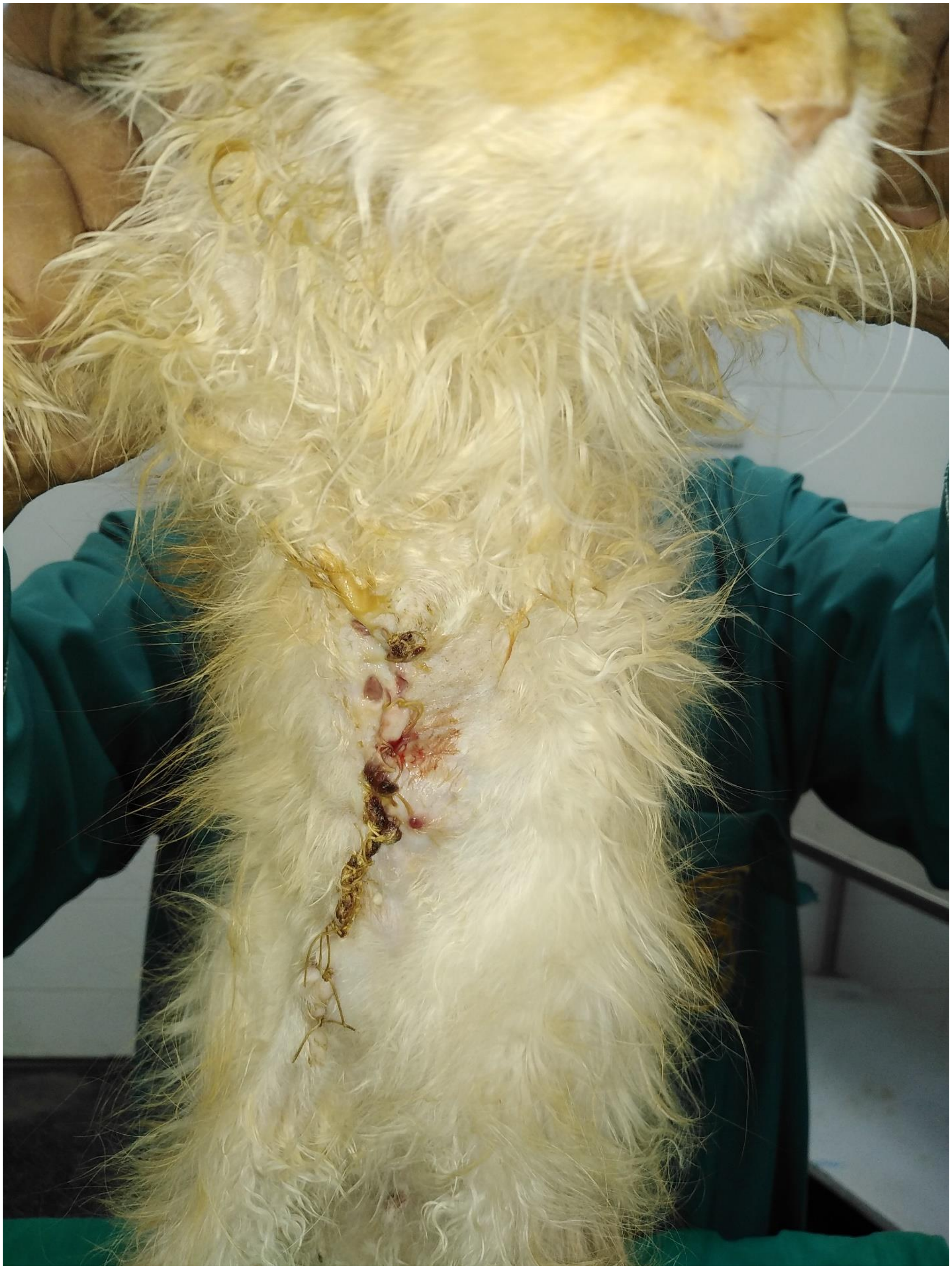

Figure 9. Application of PPTT session in Case No. 2 of TSP group during surgery

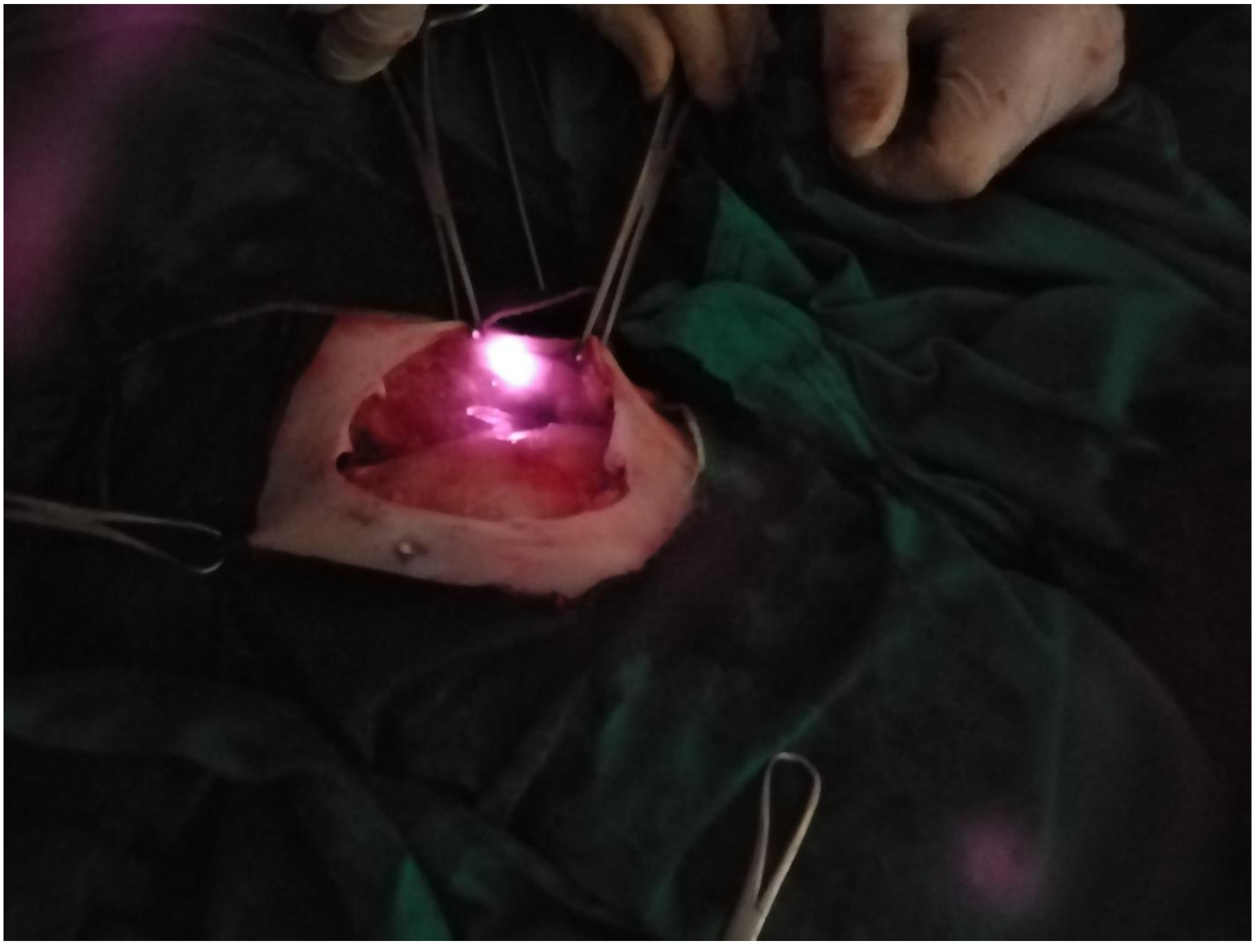

Figure 10. Case No. 2 of TSP group after 3 months of surgery

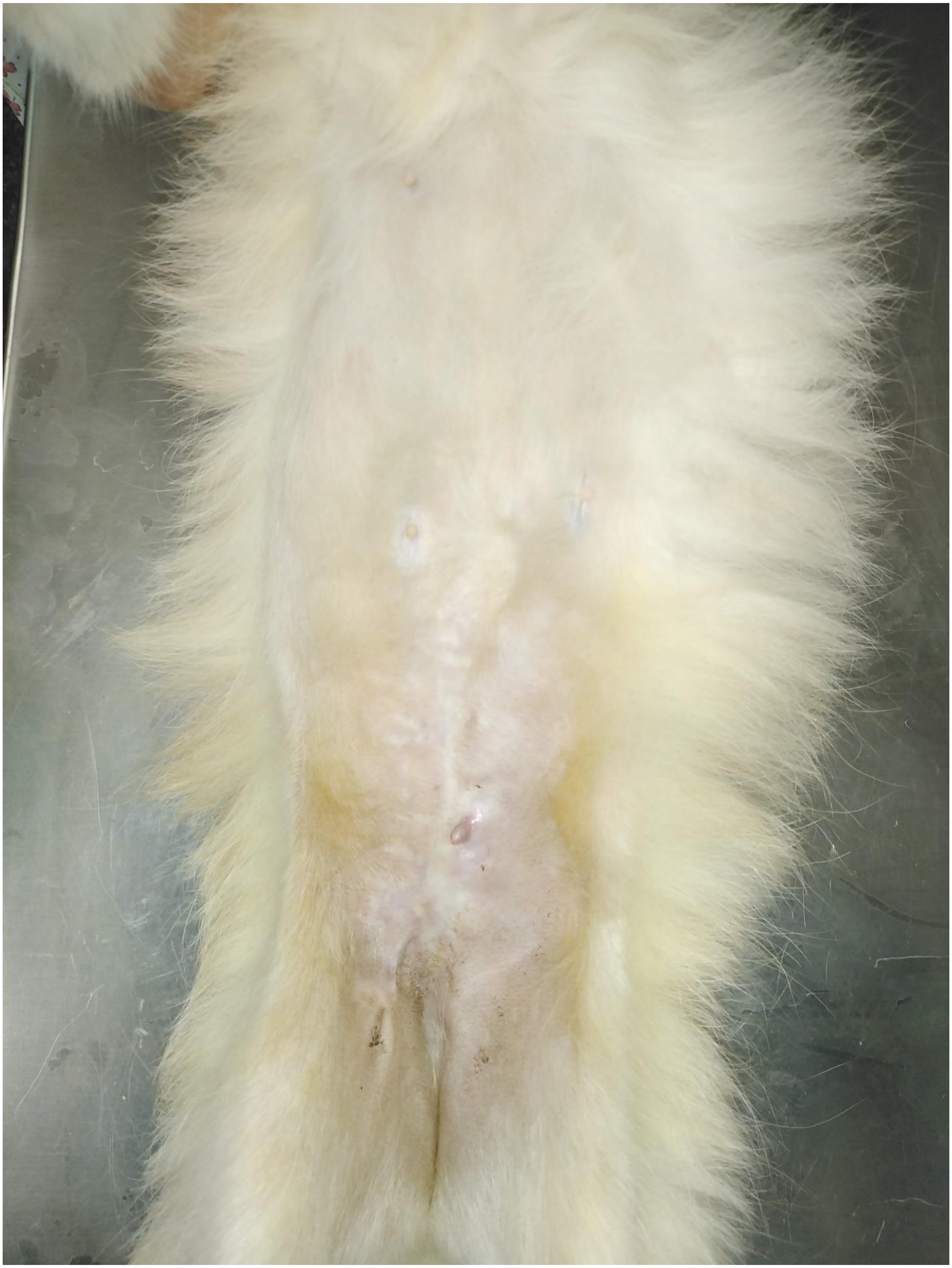

Figure 11. Case No. 6 of TSP group before surgery

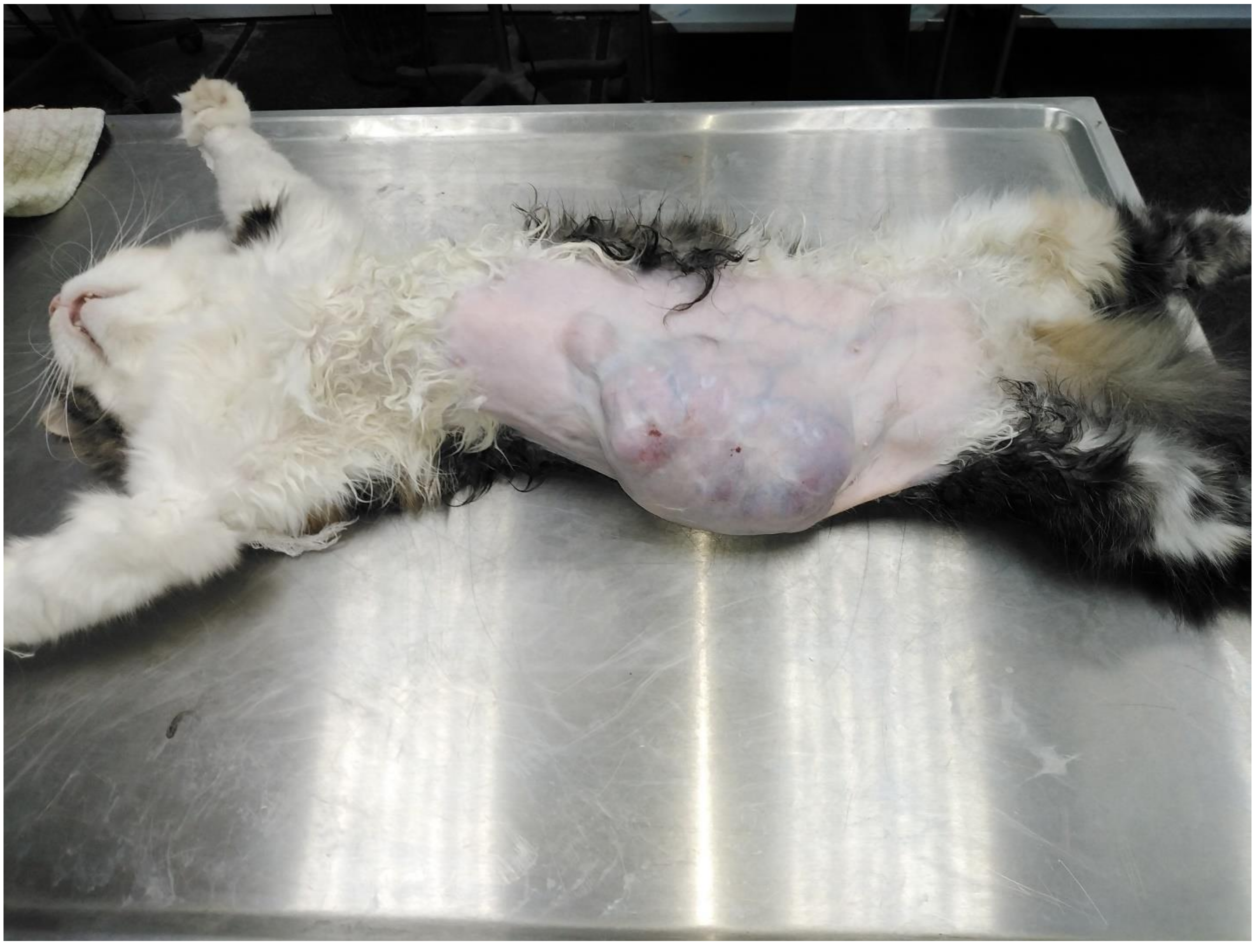

Figure 12. Case No. 6 of TSP group just after surgery

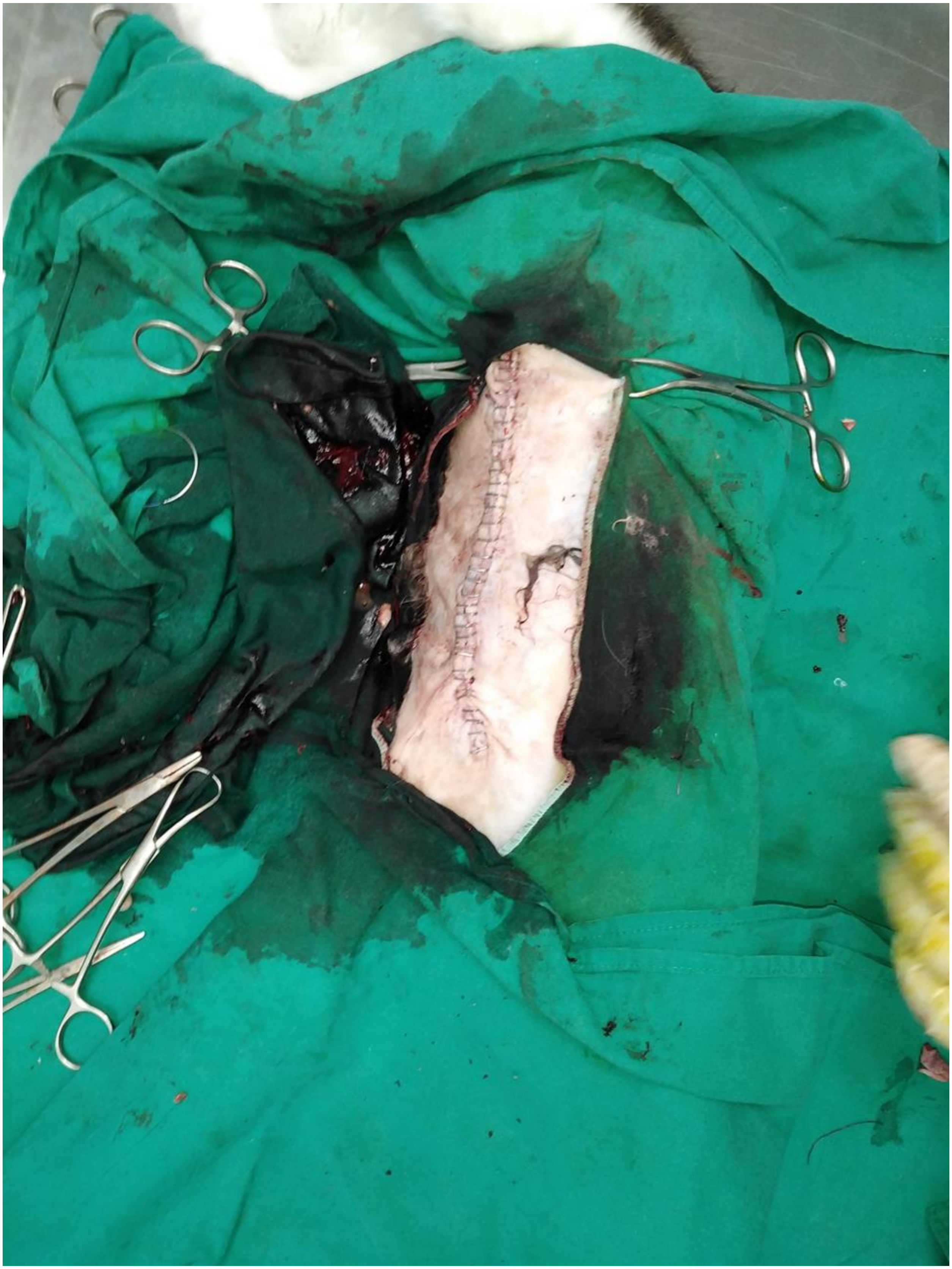

Supplement: Supplementary file 2 — Supplementary Information 2. [file 41598_2021_89894_MOESM2_ESM.pdf]
